# A multi-omic single-cell landscape of perinatal mouse skin maps lineage specification and reveals shared dynamics in human fetal skin

**DOI:** 10.1038/s12276-026-01692-5

**Published:** 2026-04-17

**Authors:** Hanjae Lee, Seunghee Lee, Seong Jin Jo, Sunhyoung Lee, Hyunjung Go, Ohsang Kwon, Jong-Il Kim

**Affiliations:** 1 Department of Dermatology, Seoul National University College of Medicine, Seoul, Korea; 2 Genomic Medicine Institute (GMI), Medical Research Center, Seoul National University, Seoul, Korea; 3 Laboratory of Cutaneous Aging and Hair Research, Clinical Research Institute, Seoul National University Hospital, Seoul, Korea; 4 Institute of Human Environment Interface Biology, Seoul National University College of Medicine, Seoul, Korea; 5 Department of Biomedical Sciences, Seoul National University Graduate School, Seoul, Korea; 6 Department of Biochemistry and Molecular Biology, Seoul National University College of Medicine, Seoul, Korea; 7 Cancer Research Institute, Seoul National University, Seoul, Korea

**Keywords:** Epigenomics, Development, Computational biology and bioinformatics, Transcriptomics

## Abstract

During the hair cycle and wound regeneration, various developmental transcriptomic features are reactivated. Consequently, studying skin development provides critical insights into both fundamental biology and skin regeneration. However, chromatin accessibility during skin development remains underexplored. To address this gap, we conducted integrated single-cell chromatin and transcriptomic analyses of developing mouse skin. Our investigation revealed key gene network axes underlying skin lineage specification. In particular, our multi-omics approach identified *Mef2c+* upper fibroblasts as putative precursor cells for smooth muscle-like appendages, such as the arrector pili muscle. Furthermore, leveraging the fetal human skin atlas, we uncovered strong cross-species correlations between mouse and human skin during development. We identified an *MEF2C+* fibroblast counterpart in human fetal skin and further delineated fibroblast lineages, including dermal sheath and arrector pili muscle, demonstrating that fibroblast developmental timelines are conserved between mouse and human. Together, this study establishes a robust, human-translatable foundation for future investigations into skin development and regeneration.

## Multi-omics approach sheds light on skin lineage specification

This study explores how skin regenerates and develops, focussing on the role of certain genes and cells. Scientists have found that adult skin regeneration is limited, but some conditions allow partial regeneration, including hair follicles. Researchers used single-cell assays to analyze chromatin accessibility and RNA sequencing to understand how skin cells develop and differentiate. They compared their findings with human fetal skin data to see whether the results apply to humans. The study identified key genes and transcription factors involved in skin development. They discovered that certain fibroblasts are crucial for forming structures such as the arrector pili muscle, which is involved in hair formation. Researchers concluded that their findings could help develop new treatments for skin repair and regeneration. Future research may focus on applying these insights to human skin healing and hair growth therapies.

This summary was initially drafted using artificial intelligence, then revised and fact-checked by the author.

## Introduction

Complete skin regeneration has long been considered unattainable in adult mice and humans. Although this goal remains a challenge, the wound-induced hair follicle neogenesis model demonstrated that partial skin regeneration, including the formation of skin appendages such as hair follicles, is achievable under specific conditions^1^. Following this discovery, accumulating evidence has revealed that transcriptomic patterns characteristic of developmental stages are reactivated during wound healing and the hair cycle^2–7^. Consequently, single-cell RNA (scRNA) studies on developing skin^8–12^ have become essential resources for understanding skin development and advancing regenerative medicine.

Single-cell assay for transposase-accessible chromatin sequencing (scATAC-seq) has been used to complement scRNA findings, particularly for multi-omics analyses aimed at characterizing gene regulatory networks and identifying lineage-driving genes and transcription factors (TFs)^13–16^. Despite its utility, scATAC analysis remains underused in research on skin development. To address this gap, we conducted time-series scATAC analysis of developing mouse skin to establish a comprehensive chromatin accessibility landscape throughout skin development. Expanding upon the scRNA map of perinatal mouse skin, our chromatin architecture and integrated multi-omics analyses validated and refined the regulatory networks governing lineage specification while uncovering novel candidate TFs and gene axes involved in fibroblast and hair follicle differentiation during skin development.

In addition, we compared our findings with the recently published human fetal skin atlas to confirm their translatability to human biology^17^. A striking cross-species correlation was found between developing mouse and human skin, with each skin component matching up to a fine level. In particular, the fibroblast lineage exhibited a conserved developmental timeline between the two species, which enabled us to further delineate specialized hair follicle-associated fibroblasts in human fetal skin, including the arrector pili muscle (APM) and dermal sheath (DS).

## Materials and methods

### Mice

All animal experiments were approved and conducted in accordance with the Institutional Animal Care and Use Committee guidelines at Seoul National University Hospital (Approval No. 22-0166-S1A1, Seoul, Korea). Adult pregnant C57BL/6 female mice (8 weeks old), with verified mating schedules in which embryonic day (E) 0.5 was defined as the day after mating, were obtained from Koatech (Pyeongtaek, Korea).

### Single-cell suspension preparation

Single-cell suspensions were prepared following the protocols described in our previous study^18^. Full-thickness back skin was harvested from the euthanized mice. The epidermis was separated from the dermis by incubating the skin with 1 mg/ml Dispase II (Roche) at 37 °C for 30 min to 2 h. The dermis was then digested with 0.125 mg/ml Liberase TL (Roche) at 37 °C for 30 min, whereas the epidermis was treated with 0.25% trypsin (Gibco) at 37 °C for 15 min. Dissociated cells were sequentially filtered through 100-μm and 40-μm strainers. The epidermal and dermal cells were subsequently combined in a 1:1 ratio.

### Single-cell sequencing data generation and acquisition

scATAC-seq data were generated for time points E18.5, postnatal day 0 (PD0), PD2, and PD4 using 10× Chromium Single-Cell ATAC reagent kits (v1 and v2). scRNA-seq data were also generated for E18.5, using the 10× Chromium Single Cell 3ʹ Kits (v3.1), following the manufacturer’s protocols at the Genomic Medicine Institute Research Service Center and Geninus Inc. (Seoul, Korea). Replicate data sets were generated for these time points. In addition, two sets of raw scRNA-seq FASTQ data for PD0, PD2, and PD4 from a previous study^18^ were reprocessed and reanalyzed. Publicly available scATAC-seq data sets for E13.5 and E16.5 (ref. ^19^), along with publicly available scRNA-seq data sets for E13.5 (ref. ^9^) and E16.5 (ref. ^12^), were also integrated into the analysis. Raw sequencing reads were processed using the CellRanger ATAC pipeline (v2.1.0) for scATAC-seq and CellRanger pipeline (v7.1.0) for scRNA-seq.

### scATAC-seq data preprocessing and integration

Fragment files and count matrices from scATAC-seq data were analyzed using the Signac/Seurat packages^20,21^. Quality control measures included the application of both general thresholds, such as percentile-based thresholds for peak_region_fragments and nCount_peaks, and data-set-specific thresholds, as detailed in Supplementary Table 1. Potential doublets were detected and removed using the scDblFinder package with standard parameters (aggregateFeatures=TRUE, nfeatures=25, and processing = “normFeatures”)^22^. Peak calling was performed using the callpeak function in Signac, using MACS2 (ref. ^23^). Peaks located on non-standard chromosomes or genomic blacklist regions were excluded. The data sets were then merged according to the standard Signac protocol, which involved generating a unified peak set, filtering out low-quality peaks by length, recreating peaks-versus-cell matrices, and merging all data sets using the merge function. This process yielded 45,406 cells and 338,904 peaks. For dimensionality reduction and data set integration, the PeakVI algorithm^24^ was used with default settings and batch key as each time point. Peaks present in at least 3,000 cells (39,174 peaks) were used to define the latent space, which was subsequently used for neighborhood calculations. The cells were clustered using the FindClusters function at a resolution of 0.9. The dermal papilla (DP) lineage cluster was further subclustered to distinguish between dermal condensate/DP (DC/DP) and DP clusters. Unless otherwise specified, normalized counts generated using the RunTFIDF function were used for the downstream analyses. All data processing and analyses were conducted on a computing server at the Genomic Medicine Institute Research Service Center.

### scRNA-seq data preprocessing and integration

scRNA-seq analysis was performed using the Scanpy interface^25^. Initial filtering of low-quality cells followed the median absolute deviation (MAD) approach^26^. Cells exceeding the thresholds of five MADs for log1p_total_counts, log1p_n_genes_by_counts, and pct_counts_in_top_20_genes, as well as three MADs for pct_count_mt, or those with >8% mitochondrial RNA counts, were excluded. Ambient RNA contamination and doublets were removed using SoupX^27^ and scDblFinder^22^, respectively. After concatenating the individual samples, genes detected in fewer than three cells were filtered out. For downstream analysis, cells were retained based on the following criteria: genes >1000 and <7000, total RNA counts >4000 and <40,000, mitochondrial percentage <5%, ribosomal percentage >5%, and hemoglobin percentage <1%. After preprocessing, 67,558 cells were identified.

Data normalization was conducted using scanpy.pp.normalize_total function with a scaling factor of 10,000, followed by natural log transformation (scanpy.pp.log1p). Highly variable genes (*n* = 3000) were identified with scanpy.pp.highly_variable_genes function, and data integration was performed using scVI (v0.20.3)^28^. The scVI model was trained with the parameters: n_layers=2, n_latent=30, gene_likelihood = “nb,” categorical_covariate_keys as each time point and each sample, and continuous_covariate_keys = [“pct_counts_mt,” “pct_counts_ribo”]. The scVI latent space was used for neighborhood construction, followed by clustering using the Leiden algorithm at a resolution of 0.9. The DP lineage cluster was further subclustered to distinguish between DC/DP and DP clusters.

### Integration with scRNA reference, differential peak analysis, and trajectory analysis

Gene activity scores derived from scATAC-seq data were imputed using the GeneActivity function in Signac. Label transfer of scRNA-seq data to scATAC-seq data was performed using FindTransferAnchors function with the canonical correlation analysis reduction method. For the correlation analysis, cell-type prediction scores were computed using the TransferData function and min–max scaled for heatmap visualization. Differential peak analysis was conducted using the FindAllMarkers function (test.use = “wilcox,” min.pct = 0.1; two-sided Wilcoxon rank-sum test) and filtered by avg_log2FC > 1.0 and p_val_adj < 0.005. The ClosestFeature function was used to map each peak to the nearest gene. Trajectory analysis was performed using Monocle3, which is implemented within the Signac interface. Data were converted to CellDataSet format using the SeuratWrappers function as.cell_data_set. Cells were clustered using the cluster_cells function (*k* = 30, reduction_method = “UMAP”), and a trajectory graph was generated with learn_graph (use_partition=TRUE). The root cells were assigned using the order_cells function, with cells from E13.5, designated as the root.

### Motif analysis and footprint analysis

Over-represented TF motifs were identified using chromVAR^29^ implemented within Signac by running the RunChromVAR function. Motif information was retrieved from the JASPAR database^30^ using the getMatrixSet function (collection = “CORE,” tax_group = “vertebrates”). Differential motif analysis was performed using FindMarkers or FindAllMarkers function (only.pos = TRUE, mean.fxn = rowMeans, and fc.name = “avg_diff”). Cluster-specific chromVAR *z*-scores were calculated using the AverageExpression function with the “counts” layer. The data matrices were extracted using the GetAssayData function. Heatmaps displaying differential motif enrichment were generated using the pheatmap package with a color scale ranging from −10 to 10. The union of the top five motifs from each cluster was used as the *y*-axis variable for the heatmaps. Footprint analysis, which identifies potential TF binding activity, was conducted using the Footprint function, and visualization was performed using the PlotFootprint function.

### Paired multi-omic analysis using FigR

FigR analysis was performed according to the standard protocol for independently assayed scATAC and scRNA data. A SummarizedExperiment object was generated using the SEfromSignac function, as specified by the FigR developers (https://github.com/buenrostrolab/FigR/issues/18#issuecomment-1385915205). Raw peak counts were used as input for FigR, and the normalized RNA matrix was imported. To optimize computational efficiency, the RNA matrix was set to 20,000 cells.

Highly variable genes (5000) were identified for both scATAC and scRNA data sets using FindVariableFeatures function in Seurat (with the RNA layer based on gene activity scores in the scATAC data set). The canonical correlation analysis reduction was then performed using RunCCA function in Seurat to generate a shared co-embedding space.

Cell pairing between ATAC and RNA components was conducted using the pairCells function (keepUnique = TRUE). This process yielded 40,814 paired cells for subsequent analysis. Peak–gene associations were identified using the runGenepeakcorr function. DORC genes were identified based on a pvalZ < 0.05 threshold and a minimum of 10 significant peak–gene connections. The DORC accessibility scores were calculated with getDORCScores and smoothed using smoothScoresNN functions. For differential DORC score analysis, normalized counts were obtained, and the top three DORC genes per cluster were identified using the FindAllMarkers function in Seurat (test.use = “wilcox,” only.pos = TRUE; two-sided Wilcoxon rank-sum test). The cluster-specific average DORC scores were calculated using the Seurat average expression function. Heatmaps were generated using the pheatmap package, incorporating the union of the top three DORC genes from each cluster. TF-DORC gene associations were identified using the runFigRGRN function. A TF–gene association score was used to filter the network, applying a threshold of 1.2 to retain relevant associations. The top activating TFs were identified based on the number of associated DORC genes that surpassed the threshold. A network of the top 25 activating TFs and their DORC genes was visualized using Cytoscape (v3.10.3).

### In silico perturbation analysis

The standard CellOracle protocol was used for in silico perturbation analysis. The base gene regulatory network (GRN) was created using our own mouse scATAC data. The peak list and its co-accessibility scores were calculated using the Cicero function within the Signac. Transcription start sites were annotated using the ma.get_tss_info function in CellOracle followed by ma.integrate_tss_peak_with_cicero function. Motifs were scanned using the tfi.scan function (fpr = 0.02), followed by tfi.filter_motifs_by_score function (threshold = 10). The fibroblast clusters were subsetted from the original scRNA data, and a force-directed graph was generated using the scanpy.tl.draw_graph function (init_pos = “X_umap”). A CellOracle object was then created from this fibroblast subset and the base GRN using the knn_imputation function with default settings and get_links function (alpha = 10). The gene network was formed using links.filter_links function (*P* = 0.001, weight = “coef_abs,” threshold_number = 2000), followed by oracle.get_cluster_specific_TFdict_from_Links and oracle.fit_GRN_for_simulation functions (alpha = 10, use_cluster_specific_TFdict = TRUE). In silico perturbation was conducted using oracle.simulate_shift function (perturb_condition = “Mef2c,” value = 0.0, n_propagation = 3). Perturbed cell simulation was performed using oracle.run_markov_chain_simulation function (n_steps = 200, n_duplication = 5) and visualized using oracle.plot_mc_results_as_sankey function.

### In situ spatial transcriptomics data acquisition and analysis workflow

New E18.5 mouse skin Xenium (10× Genomics) data and reprocessed PD2 data, partially presented in our previous study^8^, were used. Tissue section (5-μm thick) from formalin-fixed paraffin-embedded samples was analyzed using a Xenium Analyzer at Macrogen (Seoul, Korea). Following deparaffinization and permeabilization, mRNAs were targeted using a custom 50-gene skin marker panel (Supplementary Table 2), supplemented by the 10× mouse brain panel (247 genes; https://www.10xgenomics.com/products/xenium-panels). H&E staining was performed post-analysis, and data visualization for both PD2 and E18.5 was conducted using Xenium Explorer (v3.0). The initial Xenium Analyzer data were reprocessed using the updated Xenium Ranger (v2.0), adjusting the nuclei boundary expansion to 2 μm. H&E images were aligned using Xenium Explorer.

Spatial transcriptomic (ST) data were analyzed using stLearn and Scanpy interface^25,31^. Data import was conducted using the ReadXenium function in stLearn, with filtering applied for genes and cells with a minimum count of 10. Log normalization and principal component analysis (PCA) were performed, followed by neighborhood graph calculations based on PCA. Clustering was executed using the Leiden algorithm with resolutions of 1.0 for E18.5 and 1.5 for PD2. The upper fibroblast cluster in the PD2 data set was subclustered at a resolution of 0.3 to isolate the APM cluster. Spatial trajectory analysis was performed following a standard protocol using spatial.trajectory.pseudotime (eps = 50, use_rep = “X_pca”) and spatial.trajectory.pseudotimespace_global functions. Transition genes for each pathway were identified using the st.spatial.trajectory.detect_transition_markers_clades function (cutoff_spearman = 0.05) and visualized with the pl.trajectory.transition_markers_plot function. Marker-based matrix plots were generated using the scanpy.pl.rank_genes_groups_matrixplot function (layer = “scaled,” vmin = −2, vmax=2). Annotated clusters were visualized using the stLearn.pl.cluster_plot function or Xenium Explorer.

### Cross-species integration and correlation analysis

SATURN was used for interspecies integration analysis. A processed human fetal skin atlas data set was downloaded (https://developmental.cellatlas.io/fetal-skin) and randomly subsampled to 60,000 cells to roughly match the number of cells in our scRNA data. The “annotation_fine” label from the human data set and the original label from the mouse data were used as cell-type criteria for integrating all skin components. SATURN’s default pipeline was applied with num_macrogenes = 2000 and hv_genes = 8000. The resulting integrated data set underwent PCA calculation using scanpy.pp.pca function, followed by neighborhood graph construction and UMAP visualization with default parameters. Cross-species correlation was determined across all skin components (both human and mouse) using the scanpy.pl.correlation_matrix function with default parameters (Pearson's correlation coefficients). The SATURN-trained data were further annotated with broad labels based on “annotation_broad,” and mouse labels were adjusted accordingly for additional broad cell-type correlation analysis. The same process was conducted for the fibroblast subset using more specific cell-type annotations incorporating time point information (post-conception week (PCW)7–10, PCW11–13, PCW14–16, and PCW17 for human, and individual time points for mouse). Using the same integrated data set, developmental timeline comparisons were conducted via correlation analysis on mouse and human fibroblasts grouped by time points.

### Fibroblast primary culture and siRNA knockdown experiment

Primary dermal fibroblasts were isolated from PD2 mouse dorsal skin and seeded onto 10 µg/ml nephronectin (R&D Systems, 4298-NP)-coated plates in DMEM (Welgene, Korea) at 37 °C in a humidified incubator with 5% CO_2_. Nephronectin was used to mediate adhesion of α8 integrin-positive APM cells and further stimulate expression of APM markers^32^. For gene knockdown, fibroblasts were transfected with small interfering RNA (siRNA) using jetPRIME (Sartorius, Germany), according to the manufacturer’s instructions. Briefly, 30 nM of *Mef2c* siRNA (Thermo Fisher Scientific, 4390815) was mixed with jetPRIME reagent, and a negative control siRNA (Thermo Fisher Scientific, 4390843) was used as a non-targeting control. Cells were incubated at 37 °C for 24 h following transfection, after which they were harvested for downstream mRNA expression analysis.

### Quantitative real-time PCR analysis

Total RNA was isolated from primary cultured PD2 dermal fibroblasts using RNAiso Plus (Takara Bio, Japan). cDNA synthesis was performed with 1 μg of total RNA using the RevertAid First Strand cDNA Synthesis Kit (Thermo Fisher Scientific). Quantitative real-time PCR was performed on a 7500 Real-Time PCR System (Applied Biosystems) using SYBR Premix Ex Taq (Takara Bio, Japan). *C*_t_ values for each target gene were normalized to the reference gene *36B4* to obtain Δ*C*_t_ values. Relative expression was calculated using the ${2}^{-\varDelta \varDelta {C}_{{\rm{t}}}}$ method, in which Δ*C*_t_ values were further normalized to the corresponding control group to derive ΔΔ*C*_t_. Data visualization and statistical analyses were performed using GraphPad Prism (GraphPad Software). The primers used were as follows: *Mef2c*, forward: 5′-GTGGTTTCCGTAGCAACTCCTAC-3′ and reverse: 5′-GGCAGTGTTGAAGCCAGACAGA-3′; *Myocd*, forward: 5′-TCTGCCGATGGATTCTTCCGTG-3′ and reverse: 5′-AGAGCCCATCTCTACTGCTGTC-3′; and *Itga8*, forward: 5′-CCGATTTGCTGTTCCTCGCCTT-3′ and reverse: 5′-GACCTGAGCAATGGCAGTGATG-3′.

### Immunofluorescence staining

For immunofluorescence staining, PD2 mouse dorsal skin was harvested, formalin-fixed, embedded in paraffin, and sectioned at a thickness of 7 µm. Sections were incubated overnight at 4 °C with primary antibodies against Mef2c (R&D Systems, AF6786) and Itga8 (R&D Systems, AF4076). The following day, sections were washed with PBS and incubated with species-appropriate secondary antibodies conjugated to Alexa Fluor 488 or 647 (Invitrogen), followed by nuclear counterstaining with 4′,6-diamidino-2-phenylindole (Invitrogen).

## Results

### Chromatin accessibility at the single-cell level revealed continuous changes during perinatal development

**Fig. 1:**
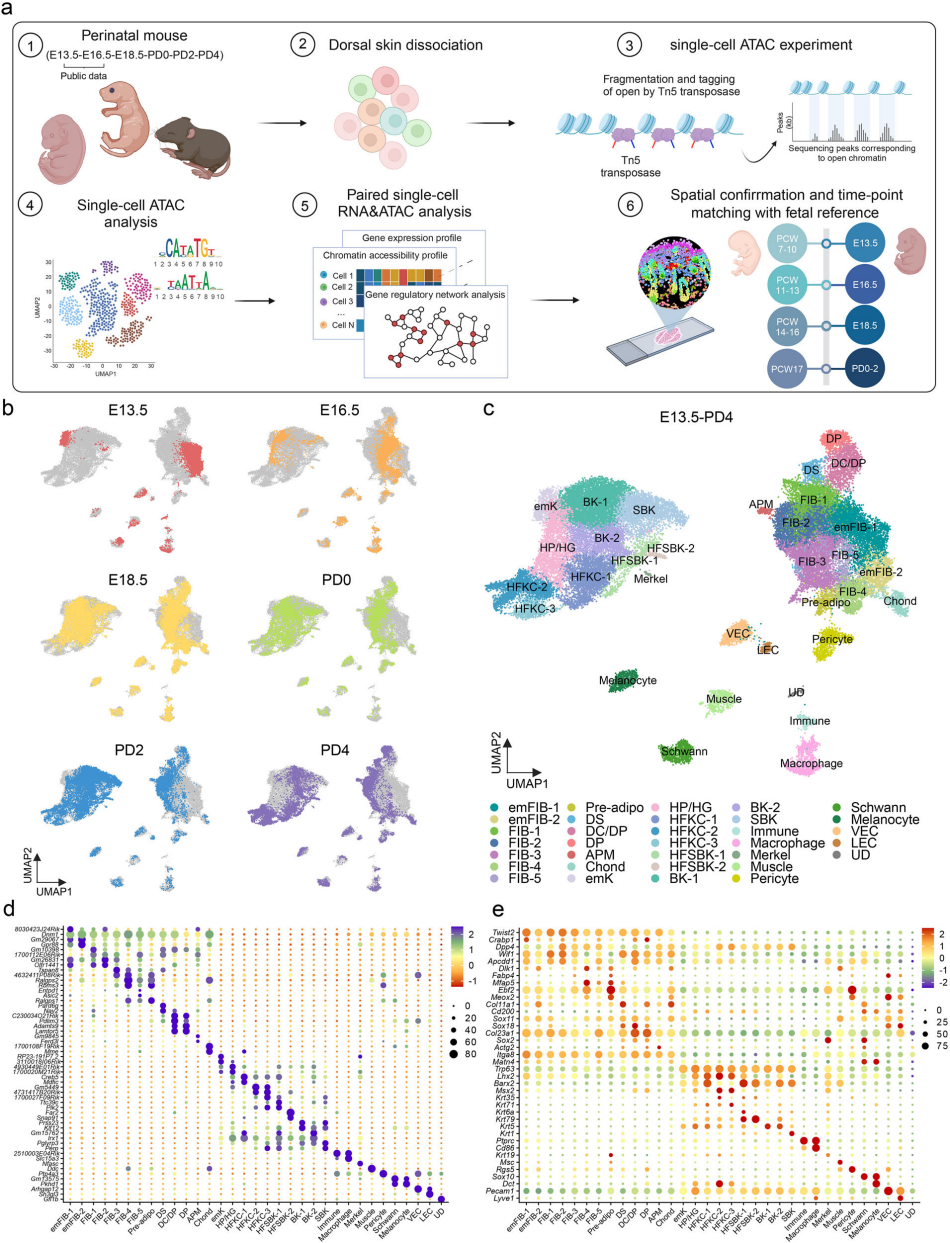
Time-series chromatin landscape of developing mouse skin. **a** Schematic representation of the study workflow. **b** Time-series Uniform Manifold Approximation and Projection (UMAP) plots of chromatin accessibility in the developing mouse skin. **c** Integrated UMAP representation spanning embryonic day 13.5 to postnatal day 4. **d** Dot plot of normalized (*z*-score) mean peak counts showing the top differential peaks for each cluster; the closest gene name is assigned to each peak. **e** Dot plot of normalized (*z*-score) mean gene activity scores of the known lineage markers. adipo, adipocyte; APM, arrector pili muscle; BK, basal keratinocyte; Chond, chondrocyte-like fibroblast; DC, dermal condensate; DP, dermal papilla; DS, dermal sheath; em, embryonic; emk, embryonic keratinocyte; FIB, fibroblast; HFKC, hair follicle keratinocyte; HFSBK, hair follicle suprabasal keratinocyte; HG, hair germ; HP, hair placode; K, keratinocyte; LEC, lymphatic endothelial cell; SBK, suprabasal keratinocyte; VEC, vascular endothelial cell; UD, undetermined.

To investigate the chromatin accessibility landscape of developing skin, we combined scATAC-seq data from E13.5, E16.5 (ref. ^19^), and 18.5, as well as from PD0, PD2, and PD4 (Fig. 1a). Preprocessing and data set integration were performed using the Signac package^20^, with dimensional reduction conducted using PeakVI^24^. Visualization of each time point on the integrated time-series scATAC Uniform Manifold Approximation and Projection (UMAP)^33^ showed a continuous and rapid transition in cell identity based on chromatin accessibility (Fig. 1b). Although scATAC data are much sparser than scRNA expression profiles, with the number of accessible peak regions far exceeding those of genes, we observed that the shift in cell identity based on chromatin accessibility closely mirrored transcriptome-based changes^8^.

We also conducted integrated scRNA analysis for the corresponding time points (Supplementary Fig. 1a) using a pipeline similar to that used in our prior work^8^. A new scRNA data set was generated for E18.5 and incorporated into the analysis. Within this integrated data set, the hair placode/hair germ (HP/HG) cluster was further refined, and the previously defined DS 2 cluster was separated into *Coch*+ fibroblasts (Coch-Fib) and chondrocyte-like fibroblasts (Chond-Fib). In addition, the *Ebf2* and *Meox2*-positive cluster (Pre-adipo), indicative of precursor cells for brown adipocyte^34–37^, were distinguished from reticular/hypodermal fibroblasts (FIB-3).

Unsupervised clustering of the integrated scATAC data set was performed (Fig. 1c). Cluster resolution and annotations were determined based on multiple criteria, including differential peak analysis (Fig. 1d), gene activity scores of known markers derived from chromatin profiles (Fig. 1e), and label transfer from the scRNA to scATAC data (Supplementary Fig. 1b). The top 10 differential peaks per cluster and their associated genes are summarized in Supplementary Table 3, and the known markers used to annotate the scRNA are depicted in Supplementary Fig. 1c and Supplementary Table 4. In addition, changes in cell-type composition across developmental time points in the scRNA data set are presented in Supplementary Fig. 1d,e.

Fibroblasts from the scRNA data were classified into distinct subtypes: two neonatal papillary fibroblasts (FIB-1, *Lef1*+/*Entpd1*+/*Apcdd1*+; and FIB-2, *Dpp4*+/*Zfp536*+)^4,8,38^, reticular/hypodermal fibroblasts (FIB-3; *Dlk1*+, *Fabp4*+)^39^, and reticular/interstitial fibroblasts (FIB-4; *Dlk1*+, *Mfap5*+)^11^ (Supplementary Fig. 2a). Correlation analysis between transcriptomic and chromatin accessibility profiles revealed strong concordance between the scRNA annotations and the unsupervised scATAC clusters, with only minor discrepancies (Supplementary Fig. 2b). An additional intermediate fibroblast population (FIB-5) was identified in the scATAC data, with weak correlations to both embryonic fibroblast (emFIB)-2 and FIB-4. For downstream analyses, we primarily focused on FIB-1 to FIB-4, which had clear correspondence with the scRNA reference. In addition, an E13.5-specific cluster, labeled as “undetermined” (UD), displayed a mixed immune and endothelial signature (Fig. 1e). Although this UD cluster may represent partial doublets, it was retained to account for the potential presence of a unique early-stage immune subset.

### Differentiation trajectories and key differentially accessible peaks for each lineage

To elucidate the differentiation direction of the integrated time-series scATAC data, we performed compositional analysis of three key components in developing skin: keratinocytes, fibroblasts, and other skin components, including endothelial and immune cells (Supplementary Fig. 3a). We then conducted a trajectory analysis using Monocle 3 (ref. ^40^) integrated within the Signac interface (Supplementary Fig. 3b). The pseudotime gradient and distinct cell fates (potential terminal cell states), represented by light gray circles, revealed key differentiation pathways. In keratinocytes, the trajectory progressed from the embryonic keratinocyte (emK) cluster through the HP/HG cluster to the hair follicle keratinocyte lineage, whereas some embryonic keratinocytes differentiated into basal keratinocytes and suprabasal keratinocytes. Similarly, emFIBs exhibited differentiation trajectories toward DP cells, upper and lower fibroblasts, and APM.

Next, we examined the differential chromatin accessibility of the well-known markers in each cell cluster. Gene activity scores revealed that the chromatin profiles of each cluster generally aligned well with its respective RNA/protein-based markers (Fig. 1e). However, when analyzed over time, some fibroblast markers, particularly *Dpp4* for upper fibroblasts and, to a lesser extent, *Dlk1* for lower fibroblasts^41^, remained relatively accessible across multiple fibroblast lineages during the perinatal period (Supplementary Fig. 3c,d). A previous scATAC study at PD0 similarly found that these markers were accessible in both upper and lower fibroblasts, suggesting that certain markers reflect cell state rather than cell type during early development^42^. Conversely, other key markers, such as *Mfap5* for myofibroblasts/interstitial fibroblasts^8^ and *Sox18* for dermal condensate^43^, were more lineage-specific (Supplementary Fig. 3e,f).

We conducted a more in-depth parallel comparison between gene expression and chromatin accessibility patterns in fibroblasts (Supplementary Fig. 4a,b), which revealed that upper fibroblast markers such as *Dpp4*, *Lef1*, *Apcdd1*, and *Entpd1* generally exhibited broad chromatin accessibility across both upper and lower fibroblast populations during the perinatal period, whereas their gene expression was more restricted to the upper fibroblasts. These patterns suggest that perinatal upper dermal fibroblasts may maintain more permissive, less lineage-restricted chromatin states, whereas specialized fibroblast lineages, such as the dermal condensate, acquire lineage-specific chromatin signatures earlier and with greater precision. It should be noted, however, that when chromatin accessibility was scaled per gene across cell types, subtle but consistent differences remained evident even among upper fibroblast markers. For example, the chromatin accessibility of *Lef1*, *Apcdd1*, and *Entpd1* was skewed toward upper fibroblast subtypes (FIB-1 and FIB-2), and *Dpp4*, though less sharply defined, was the most accessible in FIB-2, indicating that these markers can still provide clues for distinguishing fibroblast subtypes within the scATAC-seq data set (Supplementary Fig. 4c).

In parallel, many hair follicle markers showed strong alignment with the chromatin accessibility profiles. For example, chromatin accessibility of *Dkk4* (ref. ^44^), a known HP marker, was restricted to the HP/HG cluster (Supplementary Fig. 5a). Similarly, *Barx2* showed exclusive accessibility in the outer hair follicle layer (HFKC-1)^45^ (Supplementary Fig. 5b), whereas *Msx2* showed greater accessibility in HFKC-2 and HFKC-3 (inner hair follicle layers)^46^ (Supplementary Fig. 5c). HFKC-1 was also uniquely associated with *Sox9* accessibility^47^ (Supplementary Fig. 5d). Further subdivision of the inner hair follicle layers revealed that HFKC-2 was marked by *Krt35*, a cortex marker (Supplementary Fig. 5e), and HFKC-3 by *Krt71*, a marker for the inner root sheath and the Huxley and Henle layers^46^ (Supplementary Fig. 5f). A previous postnatal skin study showed that *Krt35* is highly expressed in transient amplifying cells within the hair matrix, whereas *Krt71* is strongly expressed in general matrix cells (data accessible at https://hair-gel.net)^48^. Together, the HFKC-2 cluster likely represents a more progenitor-like population within the perinatal inner hair follicle layers.

### Motif and footprint analyses elucidate lineage-driving transcription factors

**Fig. 2:**
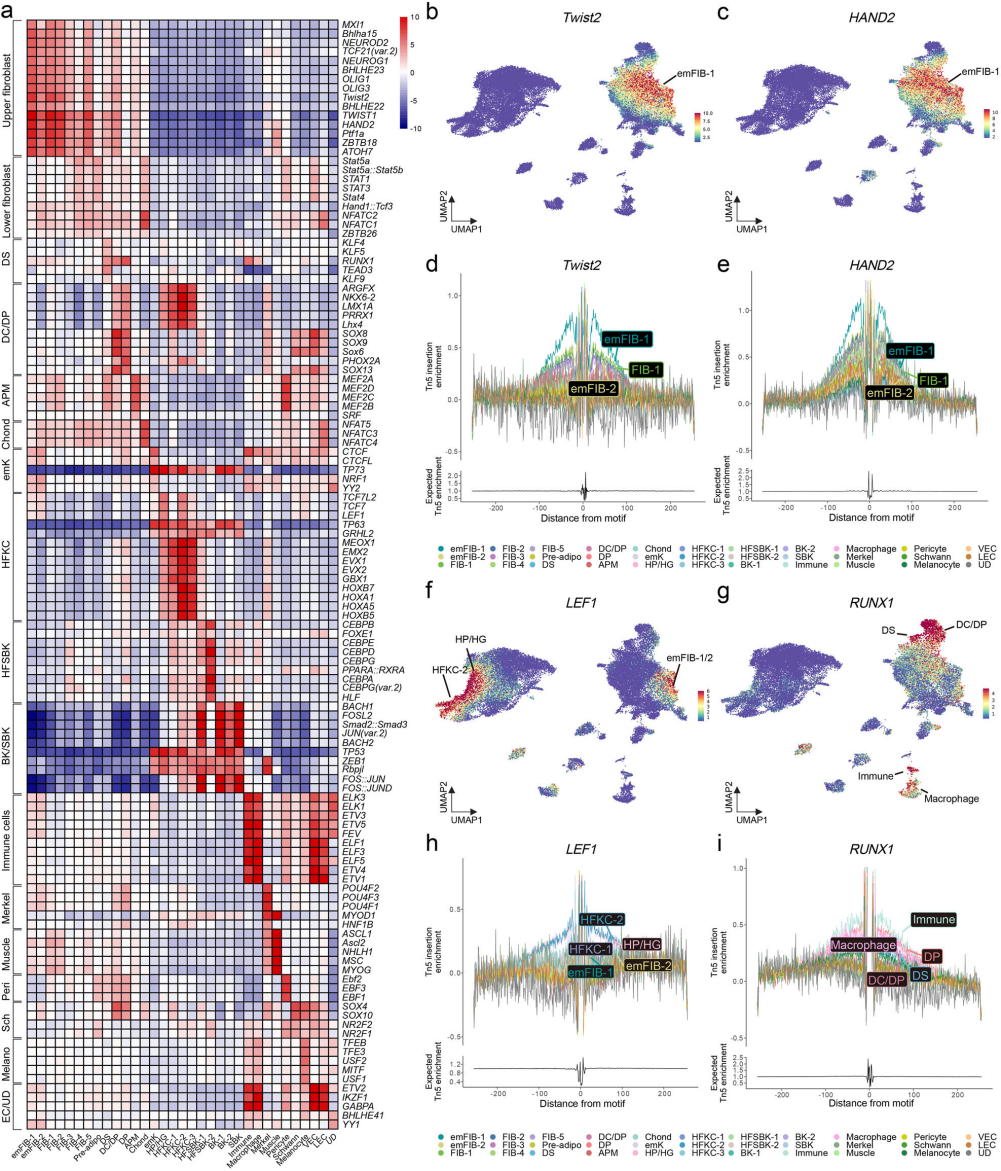
Motif and footprint analyses of the developing mouse skin. **a** Heatmap of average chromVAR *z*-scores per cluster displaying the union of the top five enriched motifs identified through differential analysis. UMAP visualization of the chromVAR *z*-scores for *Twist2* (part **b**) and *HAND2* (part **c**). Footprint plots for *Twist2* (part **d**) and *HAND2* (part **e**). UMAP visualization of chromVAR *z*-scores for *LEF1* (part **f**) and *RUNX1* (part **g**). Footprint plots for *LEF1* (part **h**) and *RUNX1* (part **i**). adipo, adipocyte; APM, arrector pili muscle; BK, basal keratinocyte; Chond, chondrocyte-like fibroblast; DC, dermal condensate; DP, dermal papilla; DS, dermal sheath; em, embryonic; emk, embryonic keratinocyte; FIB, fibroblast; HFKC, hair follicle keratinocyte; HFSBK, hair follicle suprabasal keratinocyte; HG, hair germ; HP, hair placode; K, keratinocyte; LEC, lymphatic endothelial cell; SBK, suprabasal keratinocyte; VEC, vascular endothelial cell; UD, undetermined; UMAP, Uniform Manifold Approximation and Projection.

We performed motif analysis using chromVAR^29^ to infer key TFs involved in lineage specification. Motif data were sourced from the core vertebrate set in JASPAR2020, with capitalized gene names in the figures indicating information derived from non-mouse vertebrates, primarily humans^30^. A heatmap summarizing the union of the top five differential motifs per cluster revealed that upper and lower fibroblasts could be grouped based on their motif profiles. Similarly, other dermal components, such as the DS, DP lineage (DC/DP and DP clusters), and APM, exhibited distinct motif profiles (Fig. 2a). Keratinocytes were classified into hair follicle keratinocytes and interfollicular keratinocytes. The top 10 differential motifs identified per cluster are listed in Supplementary Table 5.

Among the enriched motifs, *Twist2* and *Hand2* exhibited strong enrichment in the upper emFIB-1 (Fig. 2b, c). To corroborate motif enrichment results, we conducted footprint analysis to infer the actual TF binding activity. This analysis indicated that *Twist2* and *Hand2* indeed most frequently occupied motif sites in the emFIB-1 cluster compared with the other cell types (Fig. 2d, e). Although *Hand2* has not been extensively studied in the context of skin development, it is known to have a critical role in embryonic development, particularly within the cardiac neural crest lineage^49^. Whether upper emFIBs possess neural crest-like characteristics or are regulated by related developmental pathways remains to be elucidated.

*Lef1* exhibited enriched motif patterns in early hair follicle populations (HP/HG) and progenitor-like hair matrix cells (HFKC-2), as well as in emFIB-1 and emFIB-2 (Fig. 2f). In parallel, *Runx1* showed strong motif enrichment in the DS and DP lineage cells (Fig. 2g). Both genes also displayed concordant footprint patterns (Fig. 2h, i). *Lef1* is known to regulate both hair follicle differentiation and neonatal fibroblast development^4,50^. *Runx1* has been reported to be expressed in both DS and DP cells^51^, and its knockout in the embryonic skin mesenchyme has been shown to cause defects in hair follicle formation and maintenance^52^. Collectively, these findings demonstrate that our integrated chromatin analysis effectively captures key regulatory motifs and their binding patterns in perinatal skin.

### Paired analysis of scATAC and scRNA data highlights key lineage-driving genes and transcription factors

**Fig. 3:**
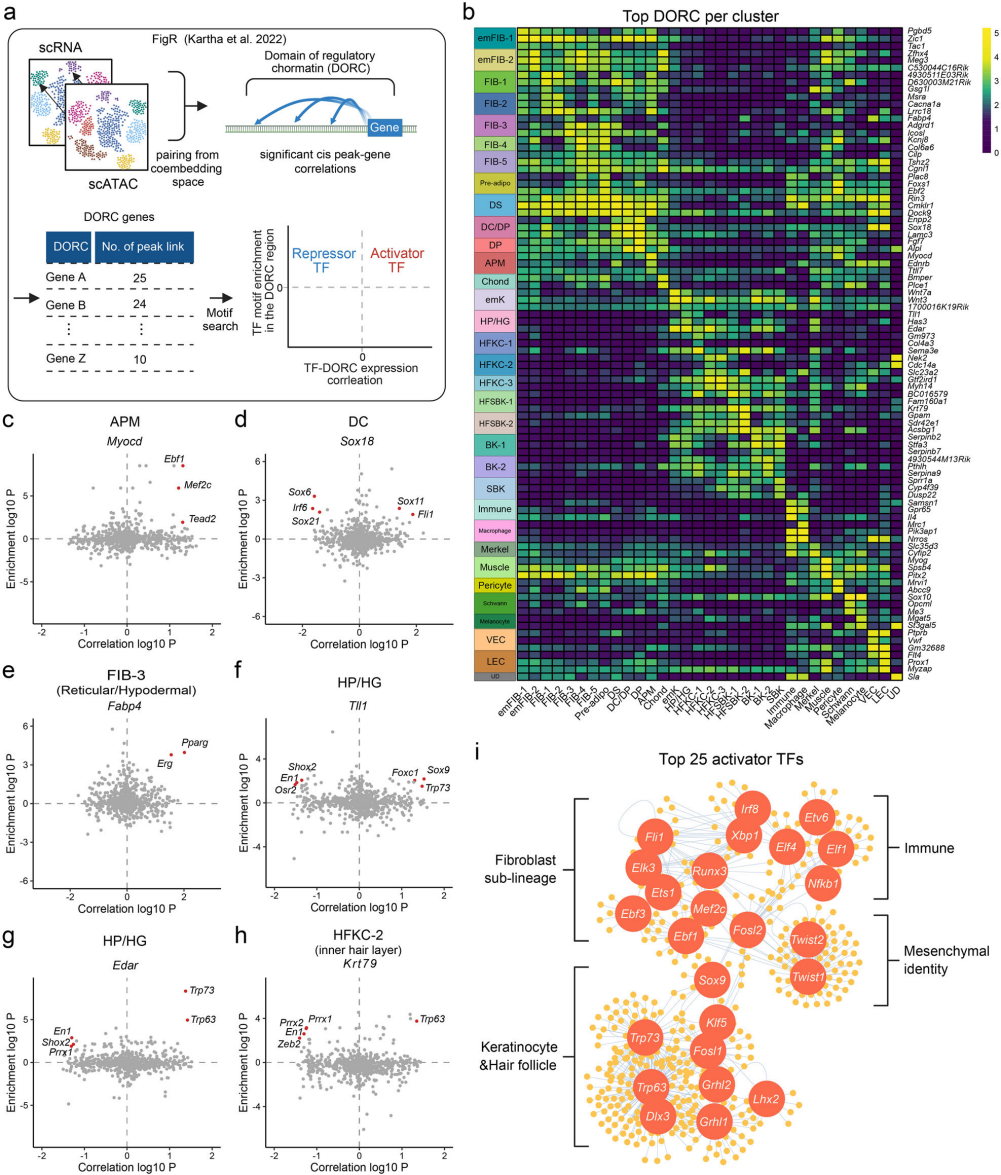
Paired multi-omics analysis of scATAC and scRNA data in the developing mouse skin. **a** Schematic of paired analysis. **b**, Heatmap depicting the top DORC genes across clusters. The color scale represents the log-normalized mean DORC score. Correlation analysis of DORC genes with enriched motifs for key DORC markers, including *Myocd* (part **c**), *Sox18* (part **d**), *Fabp4* (part **e**), *Tll1* (part **f**), *Edar* (part **g**), and *Krt79* (part **h**). The *x*-axis shows the correlation between DORC expression and transcription factors (TFs), whereas the *y*-axis displays motif enrichment within the DORC region. Red dots represent TFs with an association score of > 1.2. (**i**) Top 25 activator TFs and their downstream DORC gene network. adipo, adipocyte; APM, arrector pili muscle; BK, basal keratinocyte; Chond, chondrocyte-like fibroblast; DC, dermal condensate; DORC, domain of regulatory chromatin; DP, dermal papilla; DS, dermal sheath; em, embryonic; emFIB, embryonic fibroblast; emk, embryonic keratinocyte; FIB, fibroblast; HFKC, hair follicle keratinocyte; HFSBK, hair follicle suprabasal keratinocyte; HG, hair germ; HP, hair placode; K, keratinocyte; LEC, lymphatic endothelial cell; SBK, suprabasal keratinocyte; scATAC, single-cell assay for transposase-accessible chromatin; scRNA, single-cell RNA; VEC, vascular endothelial cell; UD, undetermined.

Expanding on chromatin architecture analysis, we adopted a systematic multi-omics approach to enhance our understanding of TFs and their downstream targets. Specifically, we used the FigR package, which integrates RNA and chromatin accessibility data to identify key genes, TFs, and regulatory networks^53^ (Fig. 3a). FigR leverages the pairing of scRNA and scATAC data to identify domains of regulatory chromatin (DORCs), gene regions characterized by high *cis*-peak–gene associations often regulated by super-enhancers^54^. We calculated DORC scores, representing chromatin accessibility at these regulatory regions, and performed differential analysis to identify lineage-specific DORC genes (Fig. 3b). For example, *Myocd* showed the highest DORC score in APM, *Fabp4* peaked in FIB-3, *Sox18* was specific to the DP lineage, and *Tll1* peaked in the HP/HG cluster. Full lists of the identified DORC genes, including the top five for each cluster, are provided in Supplementary Tables 6 and 7.

Next, we analyzed the potential TFs that activate or repress DORC genes. Gene regulatory network analysis examined enriched motifs within the DORC regions and correlated the expression of TFs with their target genes. *Mef2c* has emerged as a key activator of *Myocd*, the principal DORC gene for APM (Fig. 3c). Similarly, *Sox11* and *Fli1* were strongly correlated with *Sox18*, a crucial driver of the DP lineage^43^ (Fig. 3d). Additional findings included *Pparg* activation of *Fabp4* (Fig. 3e) and *Sox9* activation of *Tll1* (Fig. 3f). *Tll1* expression in the HP has also been reported in the previous studies^55,56^ and may have a role in early hair follicle formation. Although *Tll1* has not been extensively studied in the context of skin, it is known to function in heart development by cleaving chordin and activating BMP signaling pathways^57^. We found that *Tll1* expression, as well as the BMP signaling activity score (based on Gene Ontology term GO0030509)^58^, was highest in the HP/HG population (Supplementary Fig. 6), warranting further investigation. Moreover, *Trp73* and *Trp63* were identified as activators of *Edar*, a known HP marker^56^ (Fig. 3g), whereas *Trp63* was also linked to *Krt79*, a marker for the upper companion layer^46^ (Fig. 3h). We also ranked the top 25 activating TFs based on their number of significant associations with the DORC genes. Their regulatory network revealed distinct functional clusters of TFs and their associated genes (Fig. 3i and Supplementary Fig. 7). Key TFs with the largest number of downstream DORC genes included *Trp73* and *Trp63*, which are associated with keratinocyte proliferation and wound healing^59^; *Sox9* and *Lhx2*, linked to hair follicle keratinocyte development^60^; *Twist1* and *Twist2*, which define mesenchymal identity^61^; *Runx3*, involved in DP lineage specification^8^; and *Elf1* and *Nfkb1*, associated with immune cell regulation^62^. The full network data are provided in Supplementary Table 8.

### Spatial transcriptomics, immunostaining, and knockdown experiment support *Mef2c*+ upper fibroblasts as putative precursors of arrector pili muscle

**Fig. 4:**
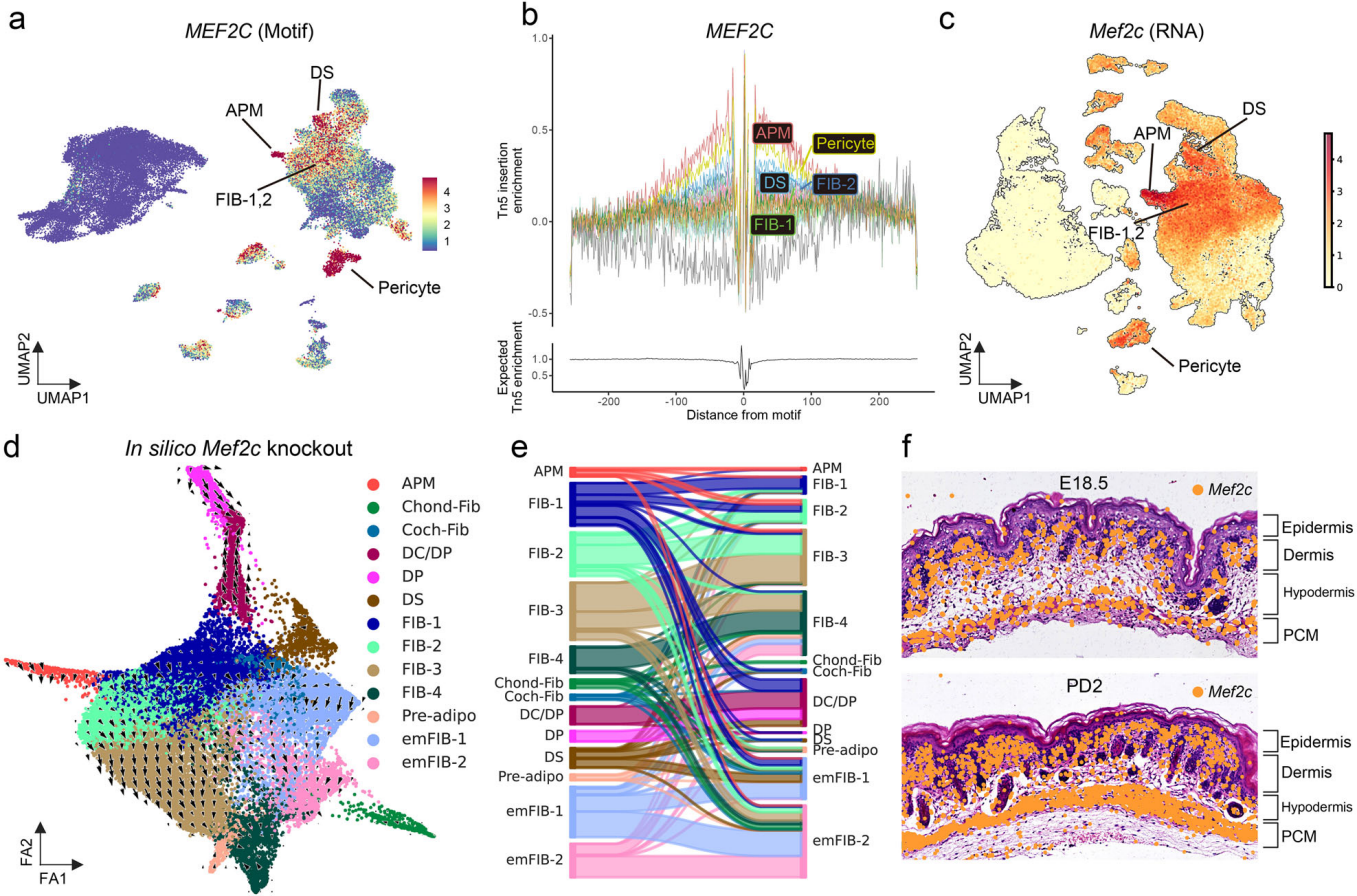
Multi-omics analysis of *Mef2c*+ upper fibroblasts. **a** UMAP visualization of chromVAR *z*-scores for *MEF2C*. **b** Footprint plot for *MEF2C*. **c** UMAP visualization of log-normalized RNA expression for *Mef2c*. **d** Force-directed graph of the fibroblast subset based on RNA expression, with each arrow depicting a simulation vector following the in silico knockout of *Mef2c*. **e** Sankey plot showing the simulated cell state transitions after *Mef2c* knockout. **f** H&E images of embryonic day 18.5 and postnatal day 2 mouse skin, with *Mef2c* expression shown as orange dots. adipo, adipocyte; APM, arrector pili muscle; Chond, chondrocyte-like fibroblast; DC, dermal condensate; DP, dermal papilla; DS, dermal sheath; em, embryonic; FIB, fibroblast; PCM, panniculus carnosus muscle; Ret/Hypo, reticular/hypodermal; UMAP, Uniform Manifold Approximation and Projection.

As the precursor cells and drivers of smooth muscle-like hair follicle components, such as APM, remain poorly understood, we further investigated *Mef2c*, a TF identified as an activator of APM. We found that the *Mef2c* motif was enriched in the APM, DS, upper fibroblasts (FIB-1,2), and pericytes (Fig. 4a). This enrichment was corroborated by the binding activity (Fig. 4b) and gene expression profiles (Fig. 4c). In silico perturbation analysis using CellOracle^63^ revealed that *Mef2c* knockout in fibroblasts impaired the development of FIB-1, FIB-2, DS, and APM (Fig. 4d,e). In addition, in situ ST analysis demonstrated that *Mef2c* exhibits a bilayer expression pattern at E18.5 and PD2, with high expression in both the upper dermis and the panniculus carnosus muscle layer (Fig. 4f).

**Fig. 5:**
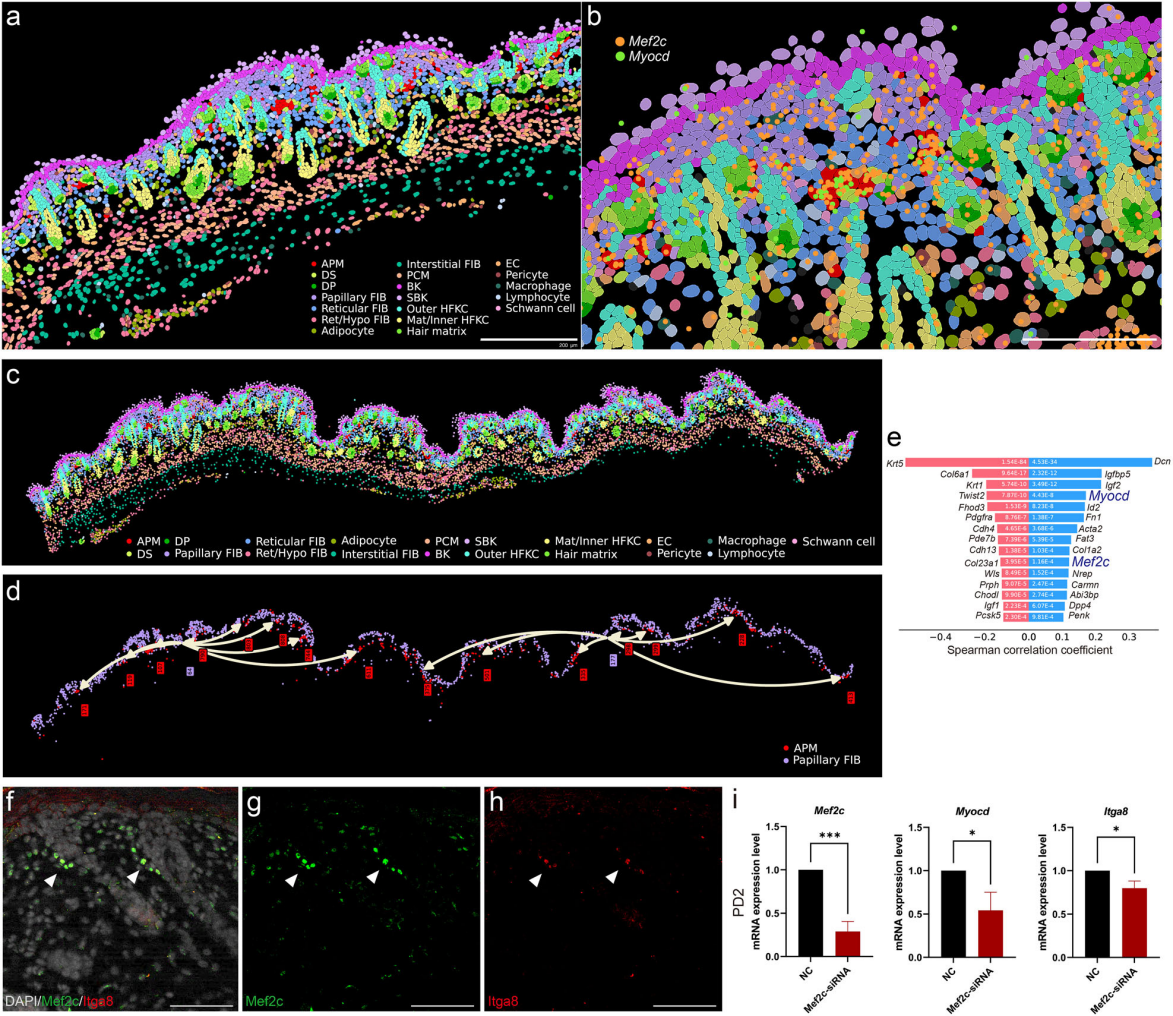
Spatial transcriptomic analysis, immunostaining, and knockdown experiment of *Mef2c*+ upper fibroblasts. **a** Cluster visualization of in situ spatial transcriptomics data on postnatal day 2; scale bar, 200 µm. **b** Zoomed-in view showing the arrector pili muscle cluster, with *Myocd* and *Mef2c* expression color-coded in green and orange, respectively; scale bar, 100 µm. **c** Cluster visualization of in situ spatial transcriptomics data of the entire skin on postnatal day 2. **d** Spatial trajectory analysis with arrows depicting transitional directions, where each number indicates subclusters within each cluster. **e** Transition genes from papillary fibroblasts (subcluster 64) to arrector pili muscle. Immunofluorescence staining of postnatal day 2 skin showing merged image of 4′,6-diamidino-2-phenylindole (DAPI), Mef2c, and Itga8 (part **f**), and individual channels for Mef2c (part **g**) and Itga8 (part **h**). Arrowhead indicates the arrector pili muscle, where Itga8 is co-stained; scale bar, 50 µm. **i**, Relative mRNA expression of *Mef2c*, *Myocd*, and *Itga8* in primary postnatal day 2 dermal fibroblasts after siRNA-mediated *Mef2c* knockdown. Data are presented as mean ± SEM (*n* = 3, two-tailed unpaired *t*-test). *P < 0.05, **P < 0.01, and ***P < 0.001. APM, arrector pili muscle; BK, basal keratinocyte; DP, dermal papilla; DS, dermal sheath; EC, endothelial cell; em, embryonic; FIB, fibroblast; HFKC, hair follicle keratinocyte; Mat, hair matrix; NC, negative control; PCM, panniculus carnosus muscle; Ret/Hypo, reticular/hypodermal; SBK, suprabasal keratinocyte; siRNA, small interfering RNA.

We further investigated ST data sets to delineate the lineage trajectory of *Mef2c*+ upper fibroblasts. Unsupervised clustering of ST data at E18.5 showed that there were no distinct APM or DS populations (Supplementary Fig. 8a–e). Although histological inspection suggested that *Mef2c*+ upper fibroblasts may cluster more closely to hair follicles at higher expression levels (Supplementary Fig. 8d), no distinct APM cells were detected within this population using either the conventional marker *Itga8* (ref. ^32^) or the newly found DORC *Myocd*, nor were spatially distinct DS cells observed (Supplementary Fig. 8d–g). By PD2, both cell types became evident, with *Mef2c*+/*Myocd*+APM and distinct DS cells (Fig. 5a,b and Supplementary Fig. 9a–e). Furthermore, spatial trajectory analysis revealed a differentiation trajectory from *Mef2c*+ upper papillary fibroblasts to APM, with *Mef2c* and *Myocd* serving as key transition genes (Fig. 5c–e). These findings suggest that *Mef2c*+ upper fibroblasts serve as putative precursors for APM formation, with *Myocd* acting as a downstream driver essential for this process. In parallel, unsupervised spatial analysis revealed spatially distinct lower fibroblasts, and trajectory analysis indicated lineage specification pathways from reticular fibroblasts to hypodermal fibroblasts, interstitial fibroblasts, and adipocytes, respectively (Supplementary Fig. 9f–k).

To further validate our findings, we conducted immunostaining for Mef2c in PD2 skin and performed siRNA-mediated *Mef2c* knockdown experiments in primary PD2 dermal fibroblasts. Immunofluorescence analysis revealed that Mef2c was localized in upper fibroblasts, with the strongest signal in APM, where the APM marker Itga8 was co-stained (Fig. 5f–h and Supplementary Fig. 10a–c). This observation corroborated the gene expression pattern identified in the spatial transcriptomic analysis. Furthermore, *Mef2c* knockdown was accompanied by downregulation of *Myocd* and *Itga8* expression in the primary dermal fibroblasts, supporting the potential upstream role of *Mef2c* in the APM formation (Fig. 5i). Although the exact mechanisms driving the transition of *Mef2c*+ upper fibroblasts into APM cells with stronger *Mef2c* expression and additional APM characteristics remain to be elucidated, prior studies on APM development have proposed that this transition is influenced by contact or signaling from the outer bulge layer of the hair follicle where the APM attaches^32,64^.

### A strong cross-species correlation was observed between developing mouse and human skin, with arrector pili muscle and dermal sheath counterparts identified in fetal skin

**Fig. 6:**
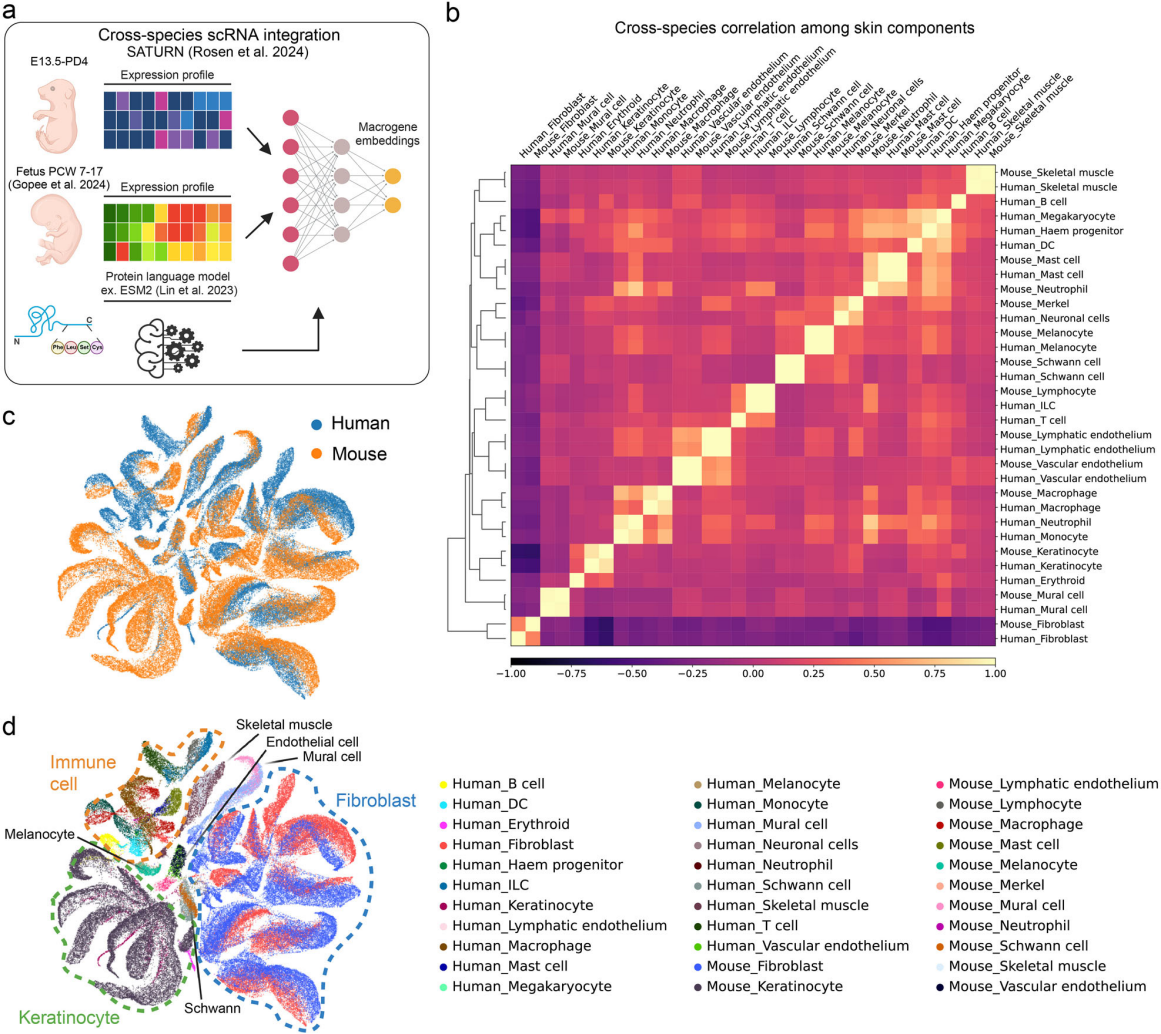
Cross-species integration and correlation between mouse and human developing skin. **a** Schematic representation of the interspecies integration workflow. **b** Matrix plot depicting cross-species correlations among all skin components. The color bar indicates Pearson's correlation coefficient. **c** UMAP displaying cross-species integration. **d** UMAP visualization of skin components from human and mouse. DC, dermal condensate; PCW, post-conception week; scRNA, single-cell RNA; UMAP, Uniform Manifold Approximation and Projection.

**Fig. 7:**
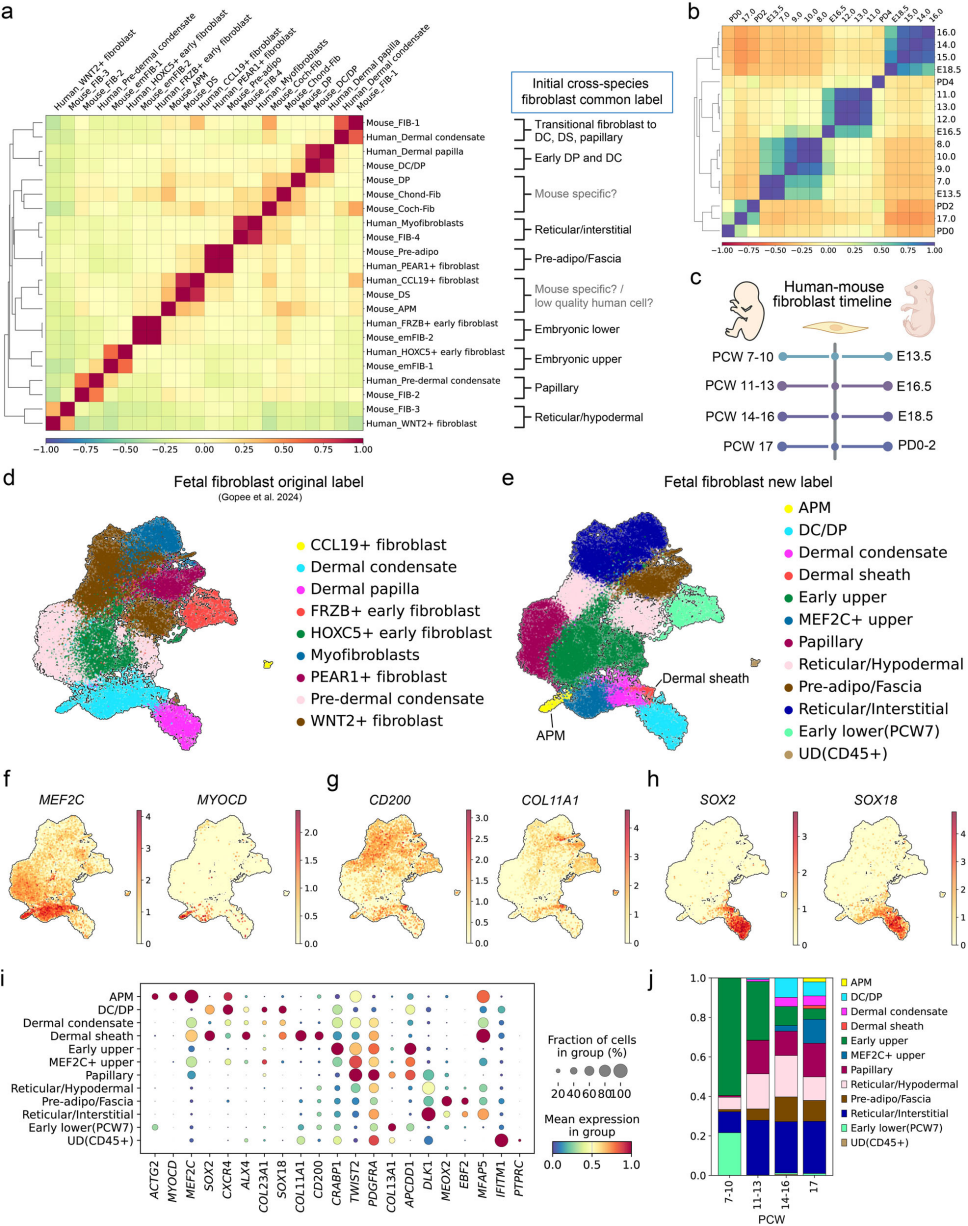
Fibroblast correlations between mouse and human in terms of developmental timeline and specific lineages. **a** Matrix plot depicting cross-species correlations among fibroblasts. The color bar indicates Pearson's correlation coefficient. **b** Matrix plot depicting correlation among fibroblasts based on their developmental timelines. The color bar indicates Pearson's correlation coefficient. **c** Schematic representation of the human–mouse fibroblast timeline. **d** Original labeling of the fetal fibroblast atlas. **e** Re-labeled fetal fibroblast atlas. Uniform Manifold Approximation and Projection plots for log-normalized expression of *Mef2c* and *Myocd* (part **f**); *Cd200* and *Col11a1* (part **g**); and *Sox2* and *Sox18* (part **h**). **i** Dot plot displaying variance-scaled log-normalized mean expression of markers used to relabel fetal fibroblasts. **j** Bar plot depicting the time-point composition of the fibroblast lineages. APM, arrector pili muscle; Chond, chondrocyte-like fibroblast; DC, dermal condensate; DP, dermal papilla; DS, dermal sheath; EC, endothelial cell; em, embryonic; FIB, fibroblast; PCW, post-conception week.

Although the mouse model provides valuable insights into human physiology, confirming the findings in human tissues remains a challenging yet necessary step. A recently published human fetal skin atlas offers an unprecedented opportunity to assess the translatability of our findings^17^. We performed cross-species integration and correlation analysis using SATURN^65^ (Fig. 6a), which revealed striking links between human and mouse among all skin components (Fig. 6b–d). Fibroblasts and keratinocytes, as well as immune cells and endothelial cells, all matched well between mouse and human. However, although a strong interspecies correlation was evident even at fine annotation levels (Supplementary Fig. 11), some fibroblast subpopulations, such as APM and DS, lacked clear human counterparts. Therefore, we further analyzed fibroblasts separately (Fig. 7a and Supplementary Fig. 12a,b), with specific time-point information incorporated (Supplementary Fig. 12c). Although certain mouse annotations, such as anagen DP, APM, and DS, did not initially match well with human counterparts, our analysis of developmental time points (Fig. 7b,c) revealed that PCW7–10 corresponded to mouse E13.5, PCW11–13 to E16.5, PCW14–16 to E18.5, and PCW17 to PD0–2. Using this temporal framework and gene markers, we could further refine the original fetal fibroblast labels (Fig. 7d–i). An *MEF2C*+ upper fibroblast population was also confirmed in human data (Fig. 7f,i), and both DS and APM formation began at PCW17 (PD0–2 in mouse) (Fig. 7j), demonstrating a strong correlation between human and mouse fibroblasts in terms of cell identity and lineage specification timing.

## Discussion

Single-cell transcriptomics have been extensively applied to studies of skin development and regeneration^3,4,8–10,12,13,46^. By contrast, single-cell chromatin accessibility data have only recently gained wider adoption in the field^42,66^. In this study, we conducted a comprehensive time-series analysis of scATAC data to map the chromatin accessibility landscape in the developing mouse skin. By integrating scATAC data with matching scRNA and in situ single-cell spatial data, we created a multi-omics framework that provides deeper insight into lineage specification and demonstrated that specific findings such as *Mef2c*+ upper fibroblasts and the formation of APM were translatable to fetal skin development.

Time-series data revealed that skin cells undergo rapid chromatin-level transitions during perinatal development (Fig. 1b,c). Differential peak accessibility (Fig. 1d), cell-type composition (Supplementary Fig. 3a), and pseudotime trajectory analyses (Supplementary Fig. 3b) were used to validate and align the cell states observed in the scRNA data (Supplementary Fig. 1). Although RNA-based and protein-based markers were generally consistent with chromatin accessibility profiles (Fig. 1e), some discrepancies were noted. For example, upper fibroblast markers such as *Dpp4* and *Entpd1* showed broader chromatin accessibility across multiple fibroblast lineages, whereas *Sox18*, an early DP lineage marker, displayed more restricted accessibility (Supplementary Figs. 3 and 4). These findings suggest that certain fibroblasts may have greater cell fate flexibility, whereas specialized lineages, such as DP, exhibit stronger chromatin-level specificity early in development.

To identify lineage-driving TFs, we conducted differential motif analysis using chromVAR (Fig. 2a). The enriched motif landscape varied significantly according to the lineage. *Twist2*, a known marker of upper emFIBs, exhibited motif enrichment exclusively in this lineage (Fig. 2b). Footprint analysis revealed an exclusive binding pattern for *Twist2* (Fig. 2d), emphasizing its critical role in maintaining the upper emFIB cell state^67^. Although the motif study, combined with pseudo-bulk footprint analysis (Fig. 2), provided deeper insights into the role of putative TFs in lineage specification, we performed additional multi-omics analysis using FigR^53^ to better inspect TF influences and their downstream targets. This approach identified DORC genes, which frequently serve as key lineage drivers with strong *cis*-peak–gene associations^54^. Gene regulatory network analysis of DORC genes and their enriched motifs further validated the key TFs regulating them. In the APM lineage, *Myocd* emerged as the top DORC gene, with *Mef2c* identified as a key activator, establishing the *Mef2c*–*Myocd* axis in APM differentiation (Fig. 3b,c). This finding aligns with previous in vitro studies that demonstrated the efficacy of *Myocd* and *Mef2c* combined with *Gata6* in reprogramming fibroblasts into smooth muscle-like cells^68^. Additionally, *Sox11* was identified as an upstream regulator of *Sox18* (Fig. 3d), which is essential for early DP lineage development^43^. Similarly, *Pparg* and its downstream target *Fabp4* formed a lineage-specific regulatory axis critical for adipogenesis (Fig. 3e).

The APM attaches to the hair follicle bulge and promotes hair follicle stem cell activation^32,64^. Clinically, the complete loss or preservation of APMs and their contact with the hair bulge have been implicated in the potential of hair regrowth in alopecia areata and patterned hair loss^69,70^. Nevertheless, neither the origin nor the precursors of APM are well understood. Therefore, we were interested in *Mef2c*, an activator TF associated with APM. *Mef2c* is well recognized for its role in cardiac development and contributes to the development of the skeletal, brain, and immune cells^71^. In humans, loss-of-function mutations in *MEF2C* have been associated with congenital heart defects^72^. *Myocd*, a downstream DORC gene of *Mef2c* in the APM, has a critical role in smooth muscle formation^73^, a function directly relevant to APM development. Motif, footprint, transcriptome, and in silico perturbation analyses indicated that *Mef2c* may be essential for the development of upper fibroblasts and APM (Fig. 4a–f). In addition, ST trajectory analysis demonstrated that *Mef2c*+ papillary upper fibroblasts are potential precursors that can differentiate into APM, during which both *Mef2c* and *Myocd* are key transition genes (Fig. 5a–e). Furthermore, immunostaining and siRNA-mediated *Mef2c* knockdown experiments supported the potential upstream role of *Mef2c* in the development of APM (Fig. 5f–i and Supplementary Fig. 10). Meanwhile, further validation is required to determine whether *Mef2c*+ fibroblasts can also serve as potential precursors of the DS lineage.

Notably, unsupervised clustering of fibroblasts revealed spatially distinct subtypes, including papillary, reticular, hypodermal, and interstitial fibroblasts, demonstrating that their transcriptomic profiles faithfully mirrored their spatial localization (Fig. 5 and Supplementary Fig. 9). In addition, trajectory pathways from reticular fibroblasts to hypodermal fibroblasts, interstitial fibroblasts, and adipocytes were confirmed, respectively (Supplementary Fig. 9f–k).

A recently published human fetal skin atlas^17^ allowed us to confirm whether our findings are translatable to the development of the human skin. We matched the fibroblast developmental timeline between the two species and further refined the original labeling of human fibroblasts. In the original study, fetal skin fibroblasts from PCW7 to 17 were compared with mouse embryonic days 12.5 to 14.5 (ref. ^11^). By contrast, our comprehensive cross-species integration analysis revealed that PCW7 to PCW17 roughly corresponded to mouse E13.5 to PD2 (Fig. 7b,c). A previous study that compared transcriptome profiles of major organs among mammals reported that human PCW7 corresponds to mouse E13.5 and human PCW16 to mouse PD3 (ref. ^74^). This further supports the general timeline correlation observed in our cross-species analysis. In addition, APM and DS were first observed at later stages of the developmental time points examined in each species (PCW17 in humans and PD2 in mice; Fig. 7j), suggesting that the overall fibroblast developmental sequence may be conserved between the two species. It should be noted, however, that alignment of cellular differentiation trajectories across species at a fine cell-type level should be interpreted with caution, as challenges remain owing to interspecies differences in developmental timing and gene expression programs, as well as technical variations such as discrepancies in sampling depth and single-cell platforms.

We previously reported that mouse upper fibroblasts lose their ability to reconstitute new hair follicles within days after birth^18^. The correspondence between fetal fibroblasts at PCW17 and postnatal mouse fibroblasts at PD2 suggests that fetal fibroblasts might begin to lose regenerative potential as early as mid-gestation. This implies that the “young” human skin that we need to understand for hair regeneration and scarless wound healing may correspond to a considerably early stage of fetal developmental. In addition, we identified *MEF2C*+ upper fibroblasts in the fetal data set and refined the original labeling, further specifying APM and DS (Fig. 7d–j). Although our ST analysis revealed that *Mef2c*+ mouse upper fibroblasts largely overlapped with the early papillary fibroblasts (Fig. 5 and Supplementary Figs. 8 and 9), the extent to which *MEF2C*+ fibroblasts overlap with papillary fibroblasts in human fetal skin remains uncertain, warranting further high-resolution spatial analyses.

Limitations of our study include differences in tissue dissociation methods between our data sets (E18.5, PD0, PD2, and PD4; Dispase II, trypsin, and Liberase TL) and the publicly available data sets (E13.5 and E16.5; trypsin for scRNA-seq and TrypLE for scATAC-seq). Given that the developmental stages themselves differ between these data sets, it is difficult to distinguish technical batch effects from true biological variation. Nevertheless, we found largely similar sets of cell types across data sets (Supplementary Figs. 1d,e and 3a), with minor exceptions such as an over-representation of muscle cells in the E13.5 scRNA-seq data and the presence of a small portion of low-quality UD cell population (UD cluster) in the E13.5 scATAC-seq data.

Our study established a comprehensive chromatin and transcriptome landscape for developing mouse skin, demonstrating the utility of scATAC sequencing and an integrated multi-omics approach to uncover lineage-specific regulatory mechanisms. We validated the transcriptomic findings, identified key gene networks driving lineage specification, and confirmed these findings with state-of-the-art ST analysis alongside cross-species comparison with human fetal skin data. This study provides a valuable human-translatable reference for understanding skin development and serves as a resource for translational applications, including the development of novel therapeutic targets for tissue repair, wound healing, and hair follicle regeneration. Finally, we provide an interactive online interface to facilitate the exploration of peaks, gene activity scores, DORC scores, and transcriptome profiles for both mouse and re-labeled human developing skin fibroblasts.

## Supplementary information

Supplementary Information

Supplementary Table 1-8

## Data Availability

Sequencing files for scATAC (E18.5, PD0, PD2, PD4), scRNA (E18.5) (GSE286328, https://www.ncbi.nlm.nih.gov/geo/query/acc.cgi?acc=GSE286328), and Xenium spatial transcriptomics data are deposited in the NCBI GEO database (GSE286129, https://www.ncbi.nlm.nih.gov/geo/query/acc.cgi?acc=GSE286129). Public data sets related to this article are accessible at https://www.ncbi.nlm.nih.gov/geo/query/acc.cgi?acc=GSE122043 (scRNA E13.5), https://www.ncbi.nlm.nih.gov/geo/query/acc.cgi?acc=GSE131498 (scRNA E16.5), and https://www.ncbi.nlm.nih.gov/geo/query/acc.cgi?acc=GSE181390 (scRNA PD0, PD2, and PD4), and https://www.ncbi.nlm.nih.gov/geo/query/acc.cgi?acc=GSE201213 (scATAC E13.5 and E16.5). An online interactive portal is accessible at https://gmi-hl.shinyapps.io/perinatal_mouse_skin_multi, and https://gmi-hl.shinyapps.io/perinatal_mouse_human_transcriptome.

## Code availability

Single-cell and spatial data were processed and analyzed using publicly available software packages. The analysis codes are available on the GitHub repository at https://github.com/hanjaelee1.
