# Supplementary material for: A multi-omic single-cell landscape of perinatal mouse skin maps lineage specification and reveals shared dynamics in human fetal skin
Source: Exp Mol Med. 2026 Apr 17;58(4):1269–83. doi: 10.1038/s12276-026-01692-5 (PMC13144478; doi:10.1038/s12276-026-01692-5)
Supplement: Supplementary file 1 — Supplementary Information [file 12276_2026_1692_MOESM1_ESM.pdf]

This PDF file includes:

Supplementary Fig. 1 to 12

Legends for Supplementary Tables 1 to 8

Supplementary Figures

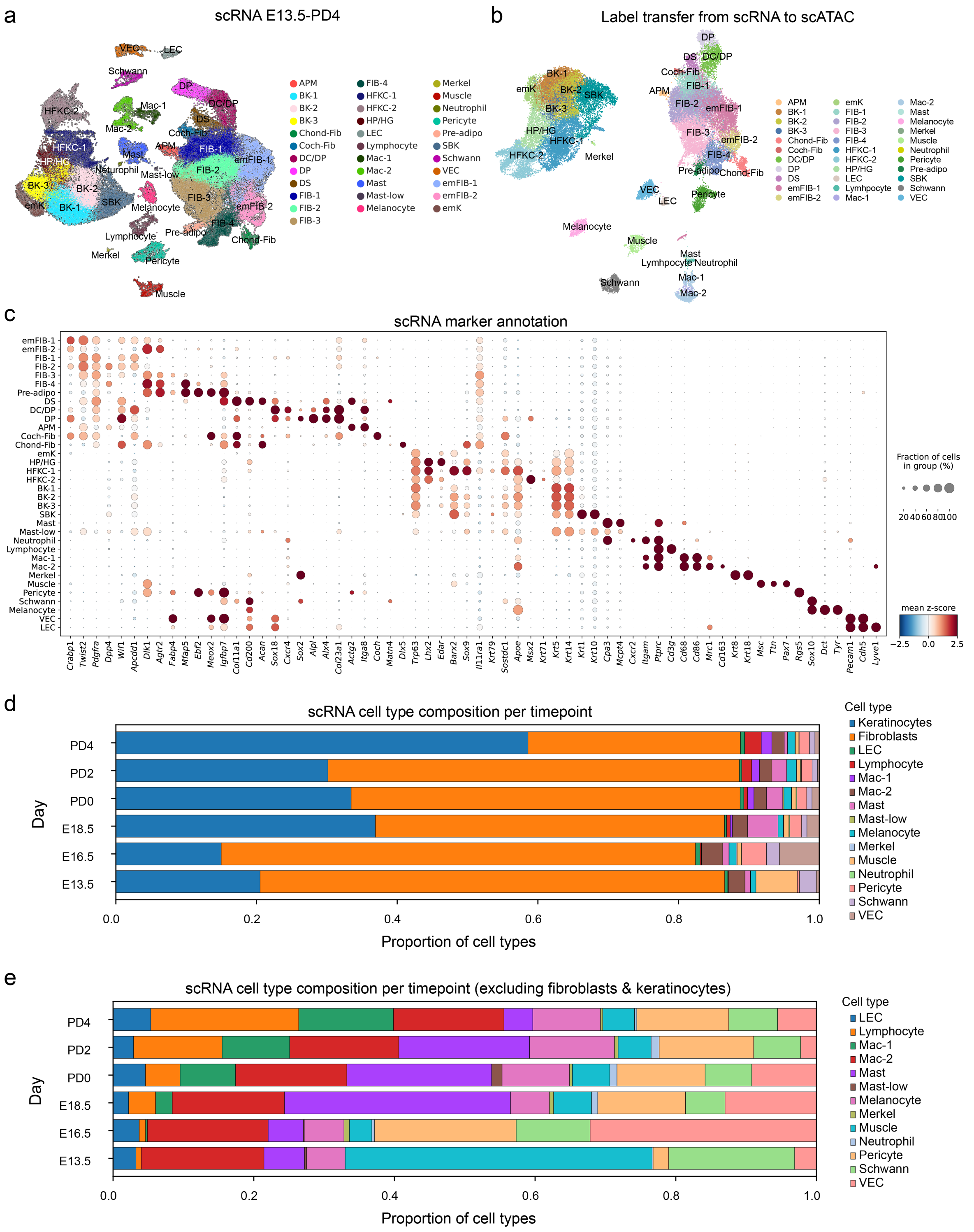

Supplementary Fig. 1. Label transfer of scRNA annotation to scATAC data.
(a) UMAP visualization of scRNA data with cluster annotations. (b) Overlay of the integrated scRNA reference onto the scATAC UMAP embedding. (c) Dot plot of normalized (z-score) mean expression of the marker genes. (d) Bar plots showing the scRNA cell-type composition of all cells (keratinocytes and fibroblasts combined) across the developmental timeline. (e) Bar plots showing the scRNA cell-type composition of non-fibroblast and non-keratinocyte populations across the developmental timeline. em, embryonic; FIB, fibroblast; DS, dermal sheath; adipo, adipocyte; DC, dermal condensate; DP, dermal papilla; APM, arrector pili muscle; Chond-Fib, chondrocyte-like fibroblast; K, keratinocyte; HP, hair placode; HG, hair germ; HFKC, hair follicle keratinocytes; BK, basal keratinocytes; SBK, suprabasal keratinocytes; Mast-low, low quality mast cells; VEC, vascular endothelial cells; LEC, lymphatic endothelial cells.

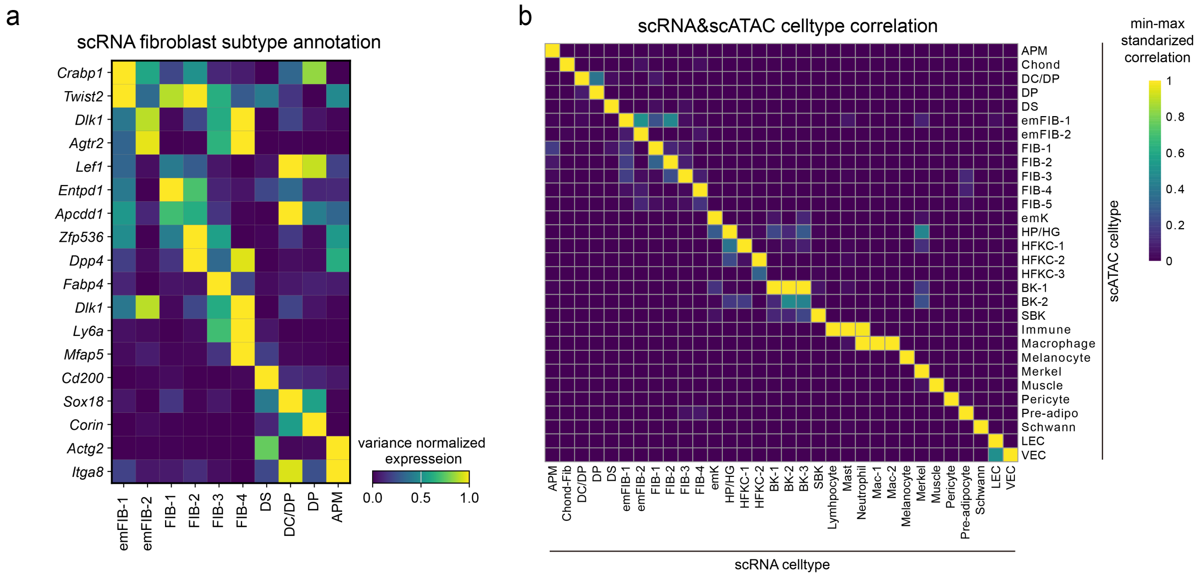

Supplementary Fig. 2. Fibroblast subcluster analysis and scRNA–scATAC correlation analysis. (a) Heatmap showing variance normalized marker gene expression across fibroblast clusters in the scRNA sequencing dataset. (b) Heatmap showing min–max scaled correlations between scRNA and scATAC clusters. em, embryonic; FIB, fibroblast; DS, dermal sheath; adipo, adipocyte; DC, dermal condensate; DP, dermal papilla; APM, arrector pili muscle; Chond-Fib, chondrocyte-like fibroblast; K, keratinocyte; HP, hair placode; HG, hair germ; HFKC, hair follicle keratinocytes; BK, basal keratinocytes; SBK, suprabasal keratinocytes; VEC, vascular endothelial cells; LEC, lymphatic endothelial cells.

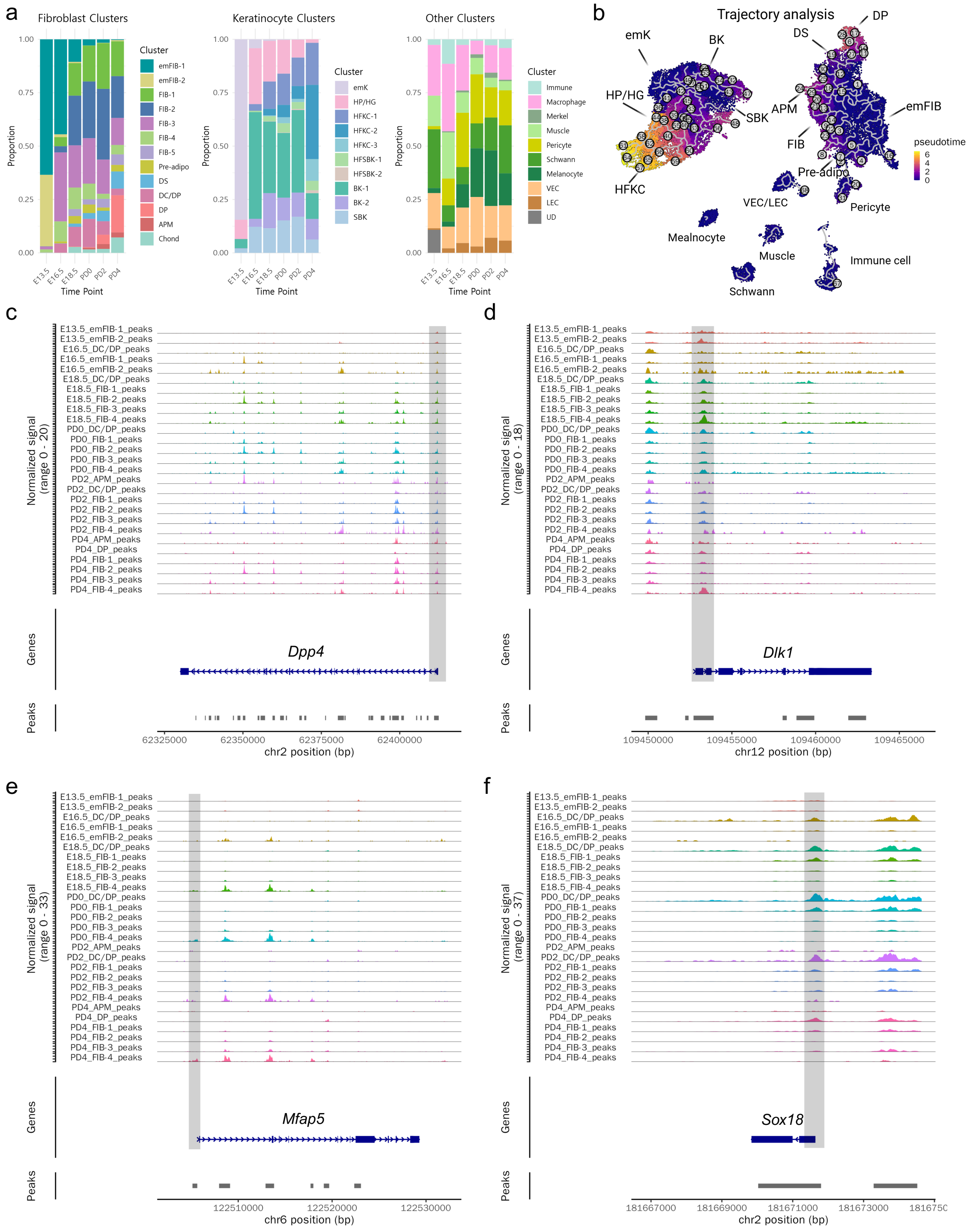

Supplementary Fig. 3. Compositional and trajectory analyses of developing mouse skin.
(a) Bar plots depicting compositional changes in fibroblasts, keratinocytes, and other skin components over time. (b) Trajectory pathway visualized on UMAP embedding using pseudotime color scale. The predicted potential cell fates are highlighted by gray circles. (c-f) Coverage plots of normalized peak counts for (c) *Dpp4*, (d) *Dlk1*, (e) *Mfap5*, and (f) *Sox18*. Gray boxes mark transcription start sites. em, embryonic; FIB, fibroblast; DS, dermal sheath; adipo, adipocyte; DC, dermal condensate; DP, dermal papilla; APM, arrector pili muscle; Chond, chondrocyte-like fibroblast; K, keratinocyte; HP, hair placode; HG, hair germ; HFKC, hair follicle keratinocyte; HFSBK, hair follicle suprabasal keratinocytes; BK, basal keratinocytes; SBK, suprabasal keratinocytes; VEC, vascular endothelial cells; LEC, lymphatic endothelial cells; UD, undetermined.

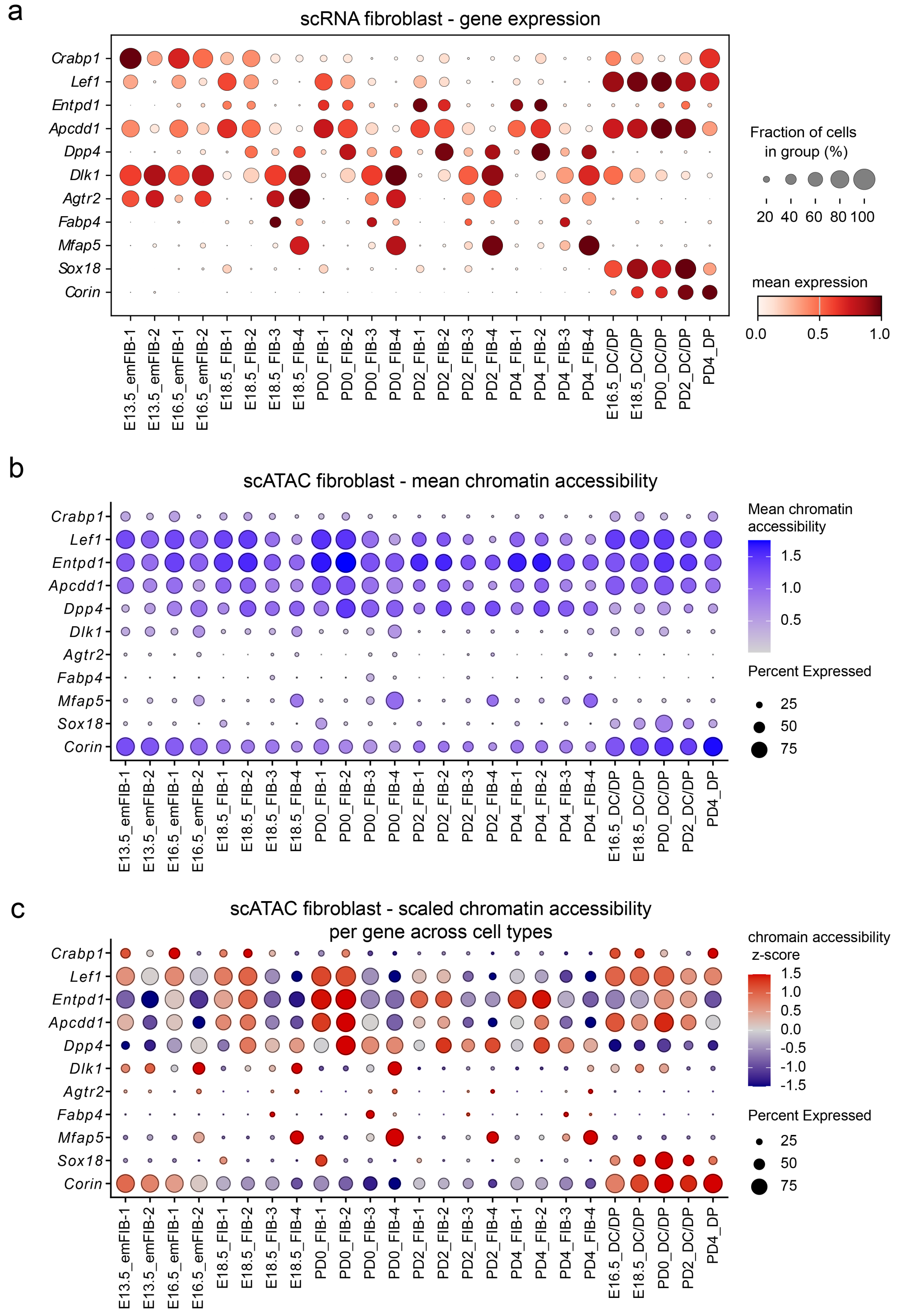

Supplementary Fig. 4. Parallel comparison of transcriptomic and chromatin accessibility profiles across fibroblast subtypes.

(a) Dot plot of variance normalized gene expression of known fibroblast lineage markers. (b) Dot plot of normalized mean chromatin accessibility (chromatin fragment counts per cell within each gene body and promoter region) of known fibroblast lineage markers. (c) Dot plot of scaled mean chromatin accessibility per gene across fibroblast cell types for known fibroblast lineage markers. em, embryonic; FIB, fibroblast; DC, dermal condensate; DP, dermal papilla

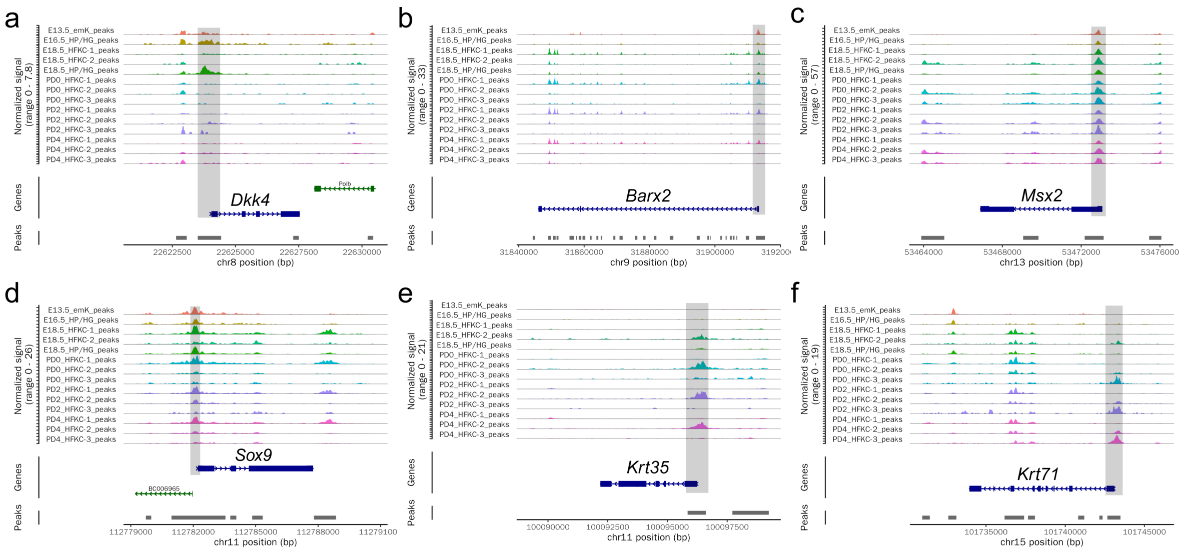

Supplementary Fig. 5. Coverage plots showing chromatin accessibility across hair follicle components. (a-f) Coverage plots of normalized peak counts for (a) *Dkk4*, (b) *Barx2*, (c) *Msx2*, (d) *Sox9*, (e) *Krt35*, and (f) *Krt71*. Gray boxes indicate transcription start sites. em, embryonic; FIB, fibroblast; DC, dermal condensate; DP, dermal papilla; APM, arrector pili muscle; K, keratinocyte; HP, hair placode; HG, hair germ; HFKC, hair follicle keratinocyte.

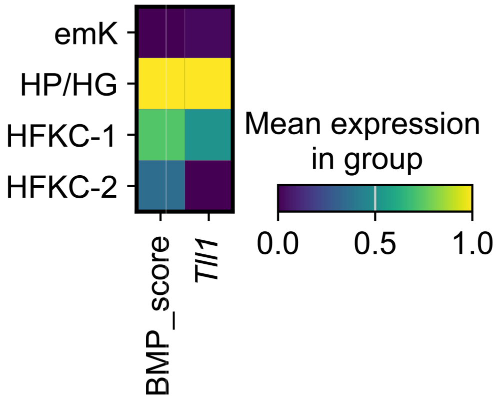

Supplementary Fig. 6. BMP signaling and *Tll1* expression in hair follicle keratinocytes.
Heatmap showing variance normalized *Tll1* expression and BMP signaling activity scores (Gene Ontology; GO0030509) across embryonic keratinocytes and hair follicle keratinocyte subtypes. em, embryonic; K, keratinocyte; HP, hair placode; HG, hair germ; HFKC, hair follicle keratinocyte.

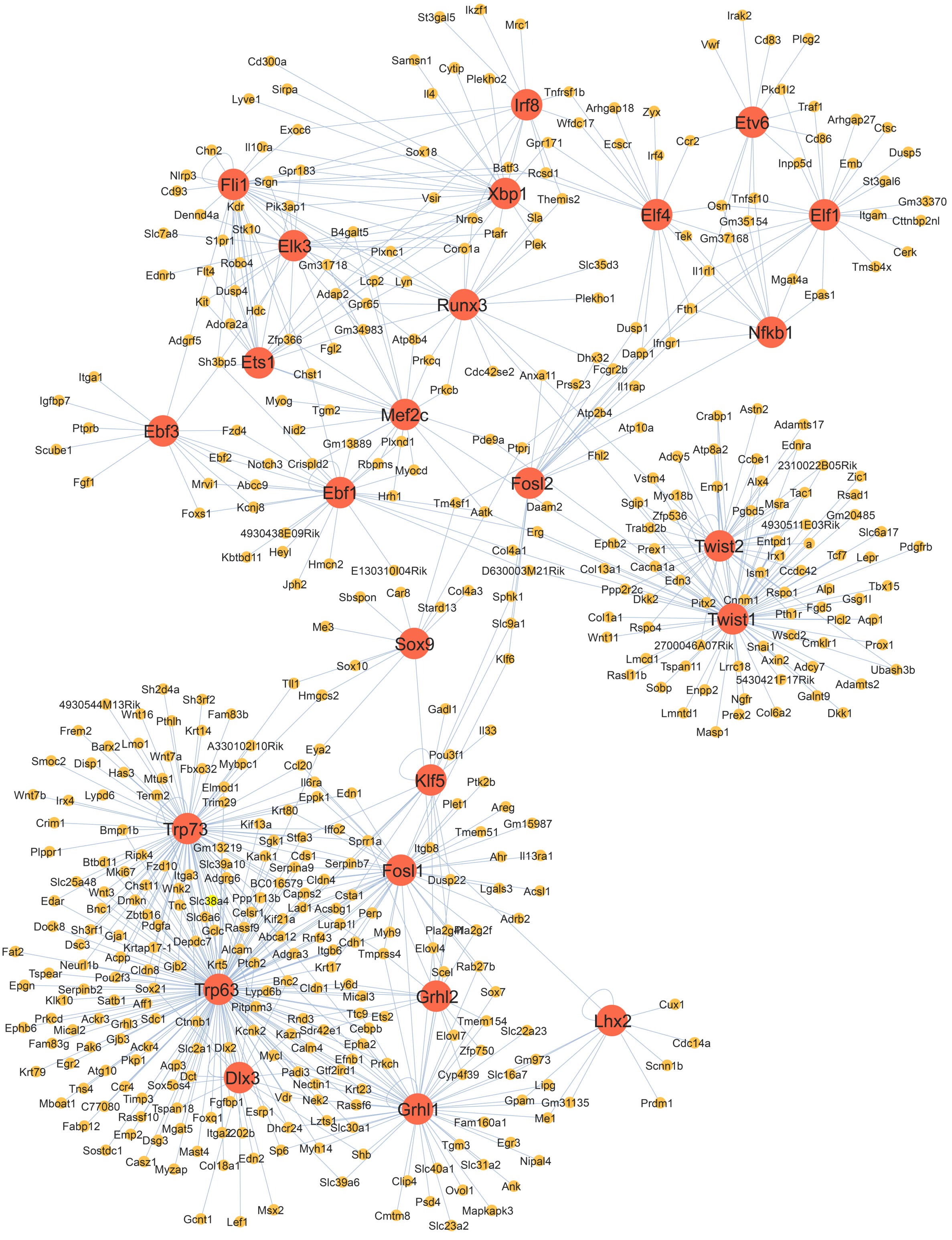

Supplementary Fig. 7. Gene network of the top 25 activator transcription factors and their downstream DORC genes.

DORC: Domain of regulatory chromatin.

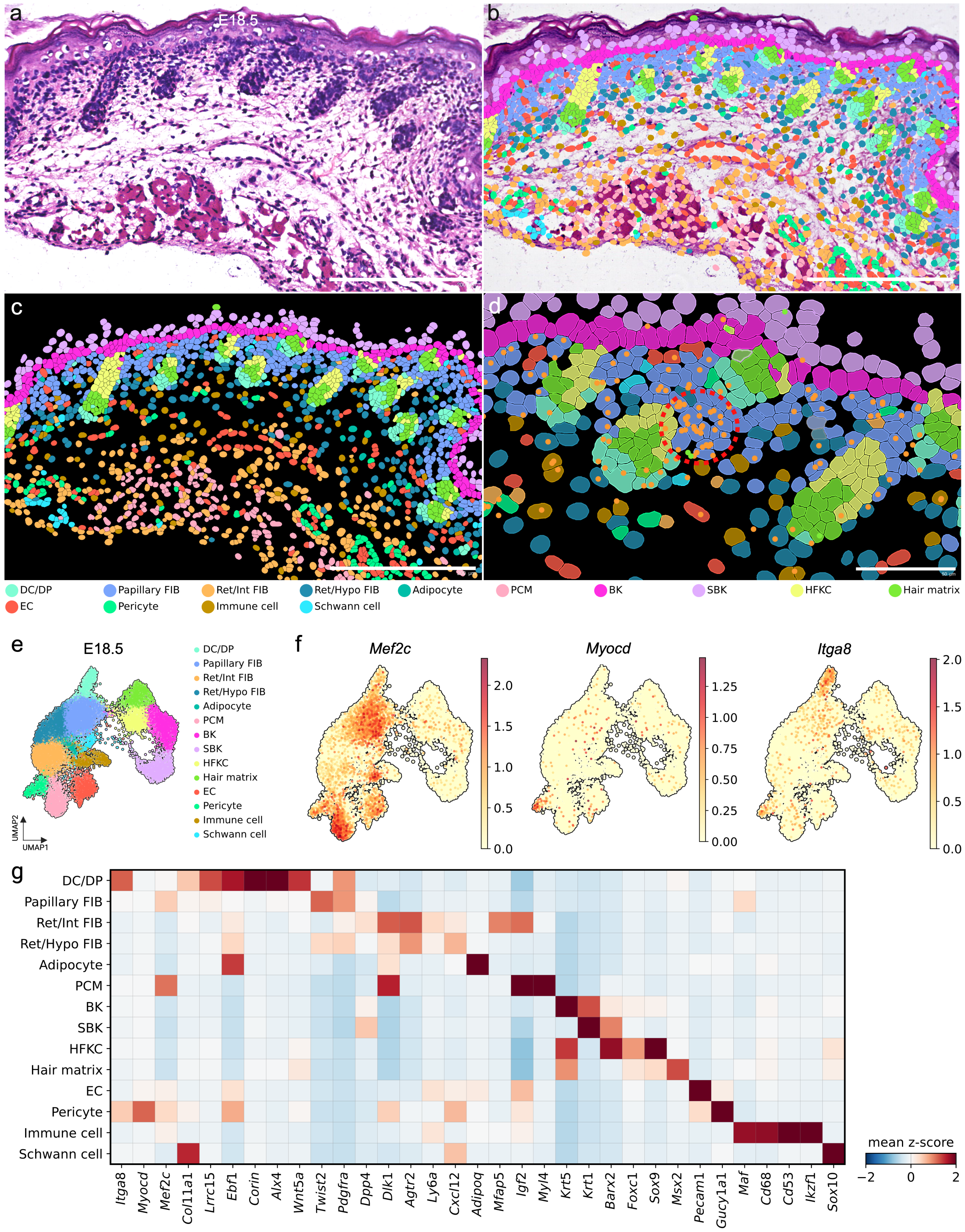

Supplementary Fig. 8. In situ spatial analysis of embryonic day 18.5 mouse skin.
(a) H&E image of the skin from E18.5. (b) Annotated cell cluster visualization overlayed on the H&E image. (c) Cluster visualization without H&E staining. (d) Magnified view of the upper fibroblast cluster with *Myocd* and *Mef2c* expression, color-coded in green and orange, respectively. The red dotted circle indicates a cluster of *Mef2c*+ cells near the hair follicles. (e) UMAP visualization of spatial transcriptomics data for E18.5. (f) Feature plots of *Mef2c*, *Myocd*, and *Itga8* gene expressions (log-normalized). (g) Matrix plot of normalized (z-score) mean expression of the known lineage markers for developing skin components. Scale bars: 200 µm for (a–c) and 50 µm for (d).

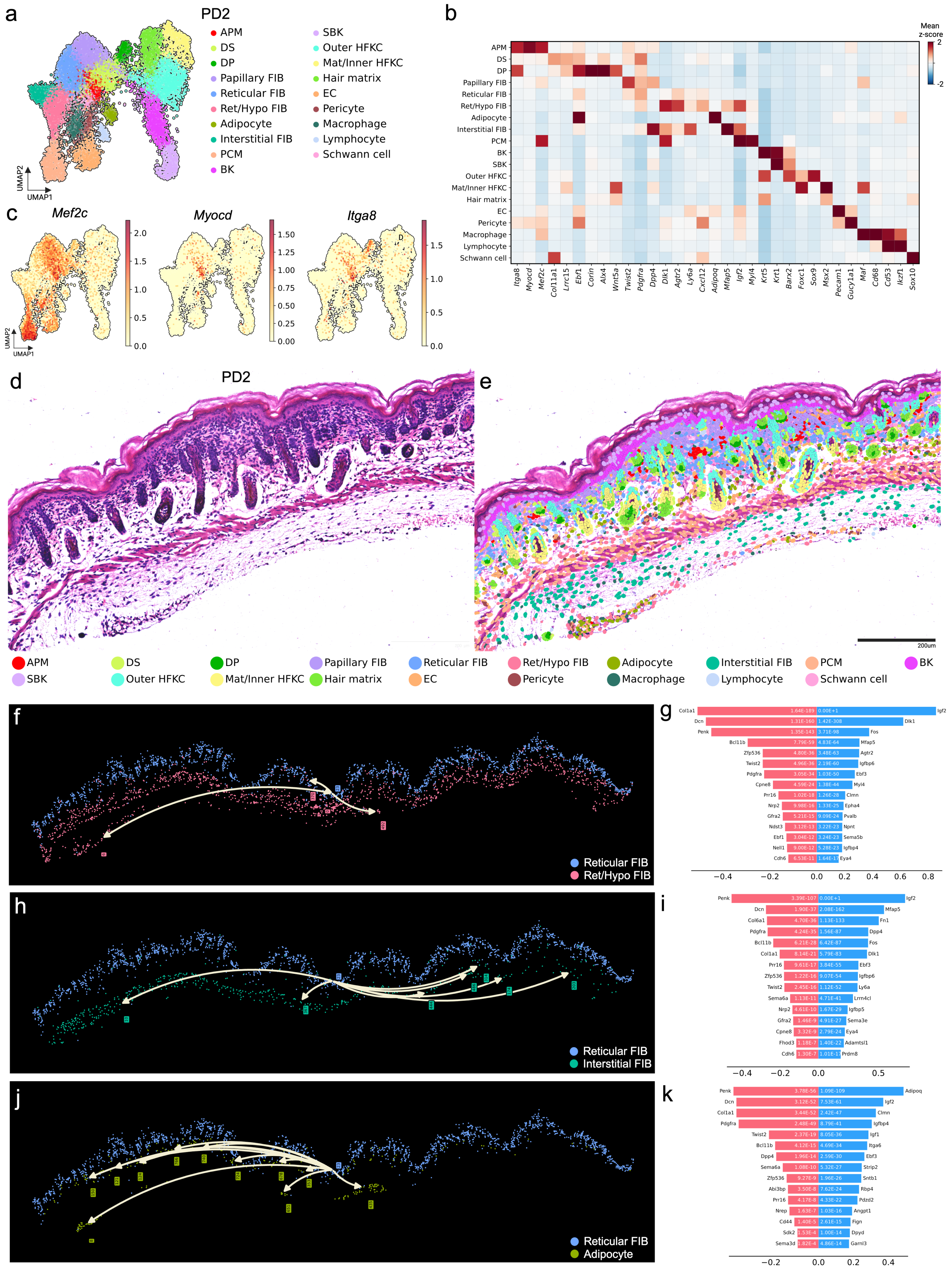

Supplementary Fig. 9. In situ spatial analysis of postnatal day 2 mouse skin.
(a) UMAP visualization of spatial transcriptomics data for PD2. (b) Matrix plot of normalized (z-score) mean expression of the known lineage markers for developing skin components. (c) Feature plots of *Mef2c*, *Myocd*, and *Itga8* gene expressions (log-normalized). (d) H&E image of PD2 skin. (e) Annotated cell cluster visualization overlayed on the H&E image. (f–k) Spatial trajectory analysis showing lineage specification pathways: (f) reticular fibroblasts to hypodermal fibroblasts, (h) interstitial fibroblasts, and (j) adipocytes. Transition genes are shown in (g), (i), and (k) for each pathway, respectively. The x-axis indicates Spearman correlation coefficient. (f,h,j) Each number in the spatial analysis indicates subclusters within each cluster.

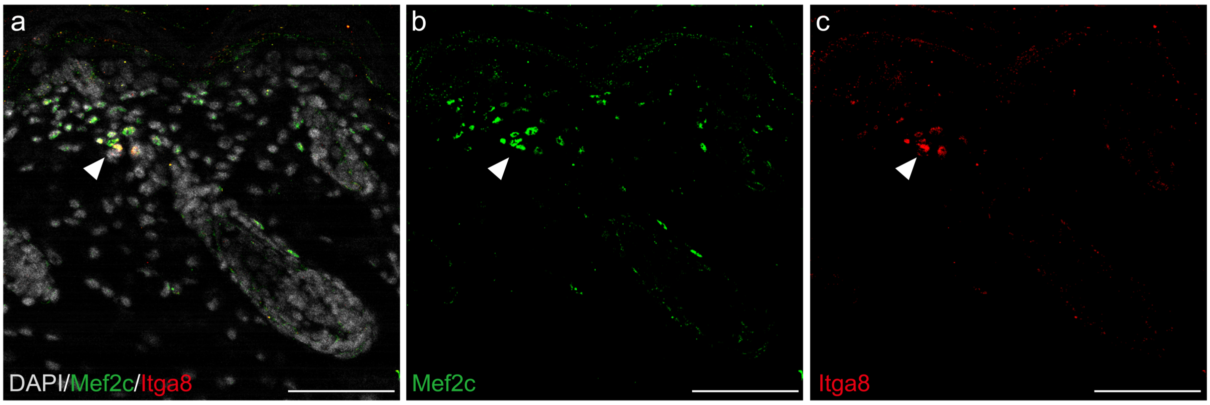

Supplementary Fig. 10. Representative immunostaining of Mef2c in postnatal day 2 mouse skin. (a–c) Immunofluorescence staining showing (a) merged image of DAPI, Mef2c, and Itga8, and individual channels for (b) Mef2c and (c) Itga8. Arrowhead indicates the arrector pili muscle, where Itga8 is co-stained. Scale bar, 50 µm.

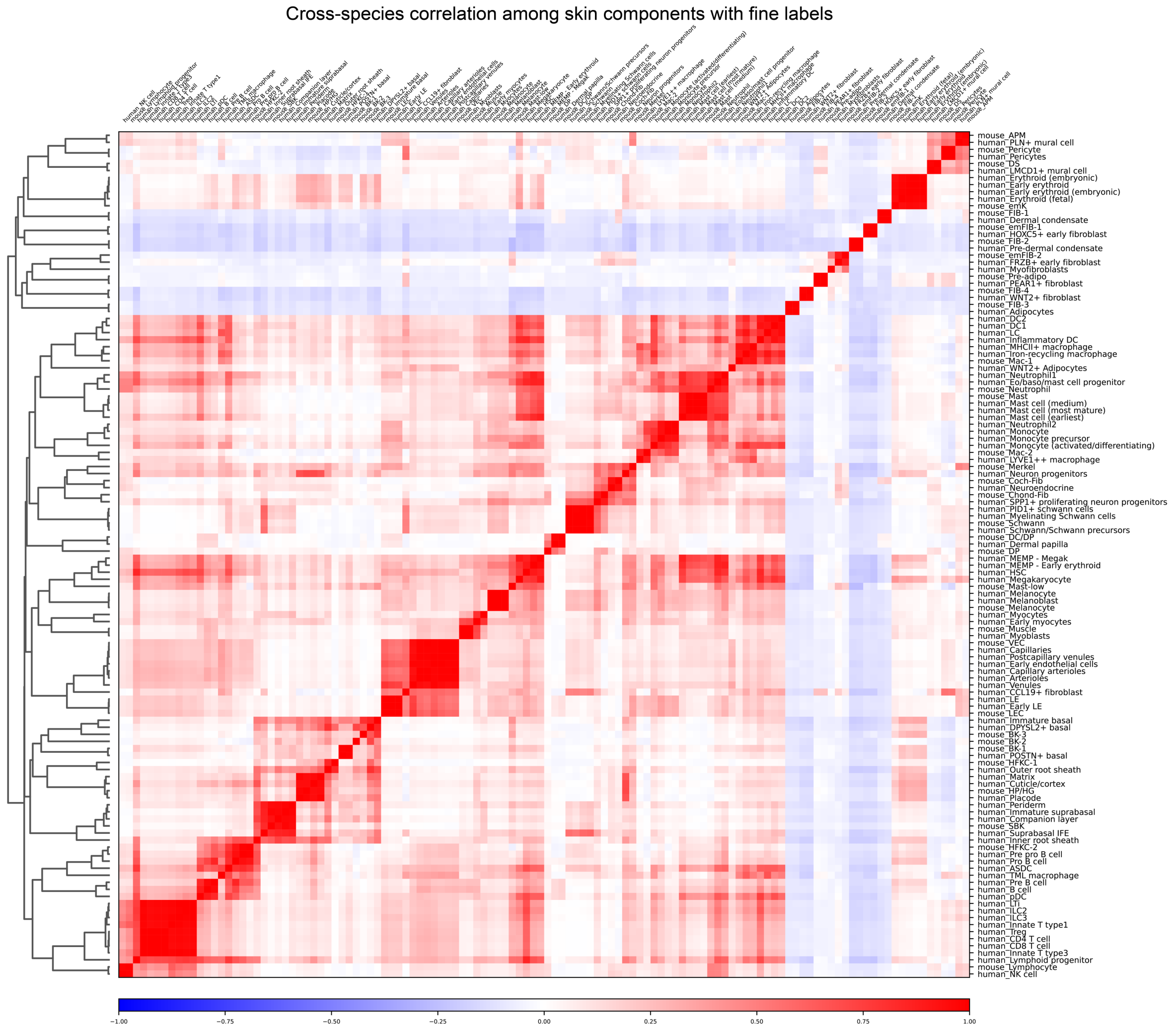

Supplementary Fig. 11. Cross-species correlations among all skin components with fine-label annotation. The color bar indicates the Pearson correlation coefficient.

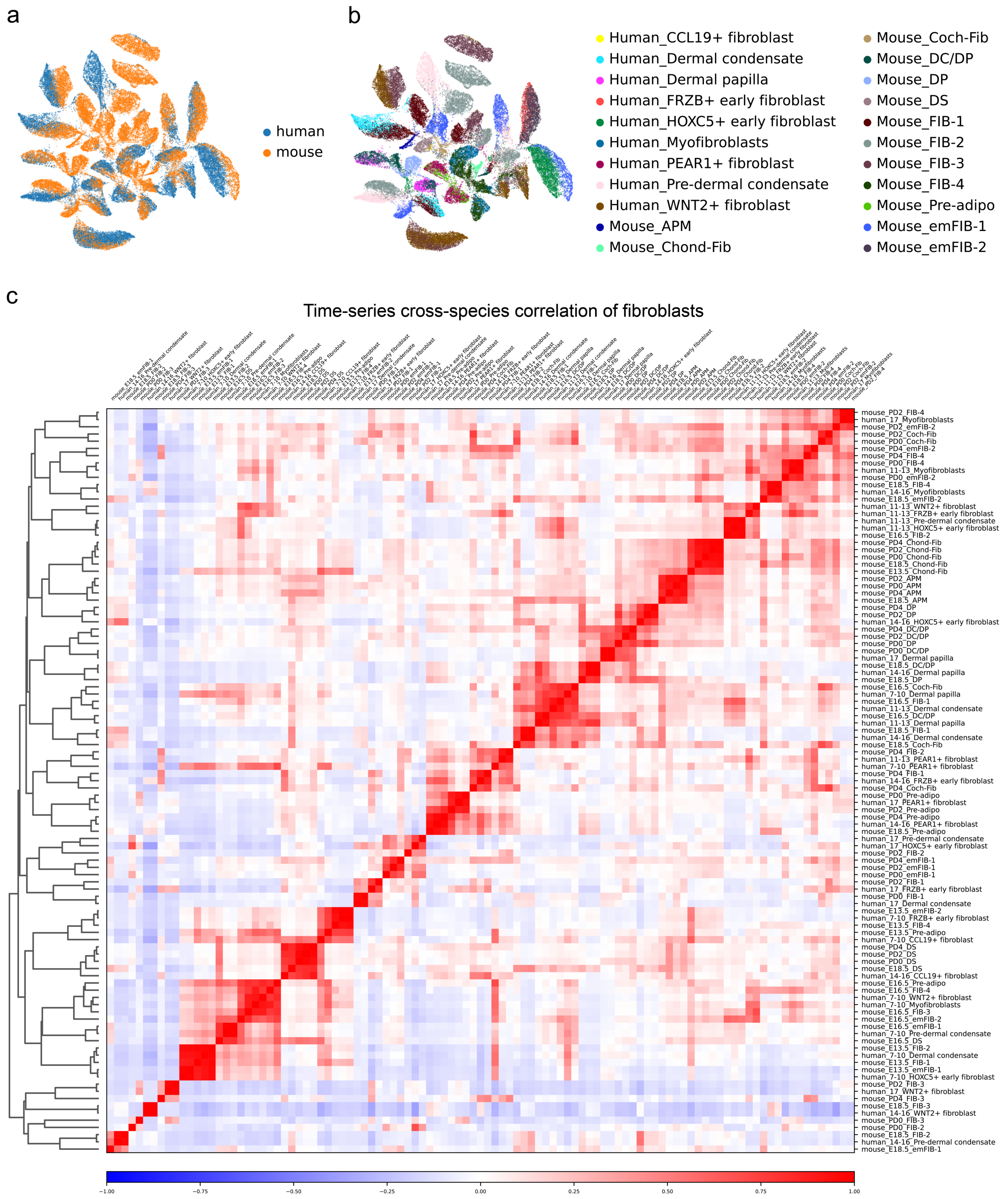

Supplementary Fig. 12. Cross-species integration and correlation between developing mouse and human fibroblasts.

(a) UMAP displaying the cross-species integration of fibroblasts. (b) UMAP visualization of fibroblast subtypes in human and mouse. (c) Cross-species correlation among fibroblast subtypes with time-point annotations. The color bar indicates the Pearson correlation coefficient.

Supplementary Tables

Supplementary Table 1. The list of quality control parameters for each scATAC dataset
Supplementary Table 2. The list of the custom 50-gene panel and Xenium brain panel used for Xenium analysis
Supplementary Table 3. The list of the top ten differential peaks and their associated genes
Supplementary Table 4. Known markers used for annotating scRNA datasets
Supplementary Table 5. The list of the top ten differential motifs per cluster
Supplementary Table 6. The list of DORC genes with thresholds of pvalZ < 0.05 and a minimum of ten significant peak-gene connections
Supplementary Table 7. The list of the top five differential DORC genes per cluster
Supplementary Table 8. The raw data for the DORC-TF network
